# Black flies (Diptera: Simuliidae) in the Galapagos Islands: Native or adventive?

**DOI:** 10.1371/journal.pone.0311808

**Published:** 2024-10-24

**Authors:** Peter H. Adler, Will K. Reeves, John W. McCreadie

**Affiliations:** 1 Department of Plant and Environmental Sciences, Clemson University, Clemson, South Carolina, United States of America; 2 C.P. Gillette Museum of Arthropod Diversity, Colorado State University, Fort Collins, Colorado, United States of America; 3 Department of Biological Sciences, University of South Alabama, Mobile, Alabama, United States of America; Instituto Leonidas e Maria Deane / Fundacao Oswaldo Cruz, BRAZIL

## Abstract

Invasive species are a threat to ecosystems worldwide, but determining if a species is adventive or native is not always straightforward. The black flies that inhabit the Galapagos Islands, long known as *Simulium ochraceum*, are blood-feeding pests of humans and livestock. They first came to the attention of residents in 1989, suggesting a recent arrival. Earlier colonization, however, has been suggested, based largely on polymorphic genetic loci. To address questions of origin, provenance, and length of residency, we conducted a macrogenomic analysis of the polytene chromosomes of the *S*. *ochraceum* complex from seven sites in the Galapagos Islands and 30 sites in mainland Ecuador, Central America, and the Caribbean. Among 500 analyzed larvae, we discovered 88 chromosomal rearrangements representing 13 cytoforms, at least seven of which are probably full species. All evidence points to a single, cohesive cytoform with full species status in the Galapagos, conspecific with mainland populations, and widely distributed in the Neotropical Region. It has an identical, nearly monomorphic banding sequence with 10 novel fixed inversions and a subtle but unique Y-linked chromosomal rearrangement across all populations sampled in the Galapagos, the mainland, and the Caribbean. We recalled the name *Simulium antillarum* from synonymy with *S*. *ochraceum* and applied it to the Galapagos black flies, and we established that *wolcotti* is a junior synonym of *antillarum*. The time(s) and mode(s) of arrival of *S*. *antillarum* in the Galapagos remain uncertain, although the wide geographic distribution, including islands in the Caribbean, suggests that the species is an adept colonizer. Regardless of how long it has been in the archipelago, *S*. *antillarum* might have assumed a functional role in the streams of San Cristobal, but otherwise has had a detrimental effect on humans and livestock and potentially on the unique birds and mammals of the Galapagos Islands.

## Introduction

As humans continue to encroach on the natural environment, the need increases to manage nature. Yet, the means to control nature also carries the power to eradicate and even cause extinction of species. Among the blood-sucking insects, for example, at least one species of simuliid is presumed extinct because of habitat alteration [1], and two genetically distinct forms, possibly species, have been driven to extinction by vector control in onchocerciasis programs [2,3]. Although eradication of invasive species can be justified and is sometimes an objective [4,5], the extinction of species cannot be ethically justified.

Oceanic islands are hotspots for biodiversity, given their isolation and consequent opportunities for speciation [6], but they are among the most imperiled ecosystems on Earth. They are, for instance, at the highest risk of any terrestrial areas for introductions of non-native species [7]. Of more than 2000 known species of insects in the Galapagos Islands, more than one-third are found nowhere else, whereas roughly one-quarter (545 species) have been introduced by human agency, of which 499 have become established [8,9].

The black fly *Simulium ochraceum* Walker has gained notoriety in the Galapagos Islands as a virulent pest—the “mosca chupa sangre” or “carmelita”—of humans and livestock, driving some subsistence farmers to shut down their operations and abandon their homesteads [10,11]. It was first reported in the islands in 1989 [12], where it breeds in streams on San Cristobal, the archipelago’s fifth largest and easternmost island, which affords permanent flowing water at its southern end [13]. Although invasive species are among the most difficult human-caused environmental problems to ameliorate [5], the Galapagos population of black flies could be suppressed or even eliminated from the archipelago by treatments with *Bacillus thuringiensis* var. *israelensis*, a larvicide specific to aquatic filter-feeding dipterans. At issue, however, is whether the population represents a native or an invasive species introduced by people within the period of human contact with the islands, roughly 500 years. Both “ancient” [14] and recent [12] introductions have been proposed; the former dictates preservation, whereas the latter opens options for management or eradication.

*Simulium ochraceum*, as currently understood, is widespread from southern Mexico and the Caribbean southward deep into Brazil and Peru [15], typically developing in small, shaded streams of hills and mountains [16,17]. It was first described from a female fly collected in an unspecified location in Mexico [18], and subsequently has figured prominently in the literature because of its anthropophily and later discovery [19] that it is a New World vector of the causal agent of human onchocerciasis, at least in the Guatemalan and Mexican foci [20]. Its structural similarity with other Neotropical species of black flies has produced a muddle of synonyms and misidentifications. Five names are currently treated as synonyms [21,22]): *antillarum* Jennings from the Virgin Islands (Saint Croix), *bipunctatum* Malloch from Peru, *pseudoantillarum* Ramírez-Pérez & Vulcano from Venezuela, *scutellatum* Lane & Porto from Colombia, and *wolcotti* Fox from Puerto Rico.

Discrepancies in its habitat, biting behavior, and vectorial capacity [20,23]; its enormous geographic distribution [15]; and its disparate taxonomic history [22] suggest that *S*. *ochraceum* consists of cryptic (i.e., isomorphic) species. The first confirmation that *S*. *ochraceum* represents a species complex came from a chromosomal investigation in Guatemala and Mexico, which revealed three cytoforms [24], later designated A, B, and C [25]. The neotype of *S*. *ochraceum* from Chiapas State, Mexico, corresponds to cytoform A [17]. Isozymes [26], cuticular hydrocarbons [27], selected mitochondrial and nuclear gene sequences [28], and additional chromosomal studies [29] support the distinction of cytoform C as a distinct species apart from A and B. DNA barcoding of samples from a wider geographic area also shows that *S*. *ochraceum* is a complex of species [30], perhaps even larger than that indicated by the earlier geographically restricted chromosomal studies [24,25].

Human onchocerciasis in the New World has largely been eliminated [31], but the pest problem in the Galapagos Islands remains. It provided the primary motivation for our investigation, driven by the need to determine if so-called *S*. *ochraceum* in the Galapagos is native or recently introduced. The high frequency of cryptic species in the Simuliidae complicates assessments of biological attributes, geographical distribution, and pest status [32], and is a matter that should be addressed.

Band-by-band comparisons of polytene chromosomes in the larval silk glands provide a potent means of studying population structure and revealing cryptic species of black flies [1], perhaps because many speciation events in the Simuliidae might be driven by chromosomal rearrangements [33,34]. The banding patterns of the polytene chromosomes also can reveal the probable source areas for insular populations [35]. We, therefore, use the polytene chromosomes in a macrogenomic (cytogenetic) study to explore the origins of the Galapagos Island population of so-called *S*. *ochraceum* and its relation to mainland populations.

## Material and methods

### Ethics statement

The Ecuadorian government (Ministerio del Ambiente) issued permits N° 006-2014-IC-FLO-FAU-DPE-MA/MAE-DPAE-2014-0627 to collect and N° 3000-006-2014-IC-FLO-FAU-DPE-MA/MAE-DPAE-2014-0596 to export samples. The Galapagos National Park Directorate provided research permit No. PC 52–14 and export permit No. 96–2014. The sample from Costa Rica was collected in support of the research of B. V. Brown and A. Borkent under the auspices of the National Science Foundation (DEB-1145890). Collections in other countries were made on public land. No collections involved endangered or protected species.

### Taxonomic scope

**Fig 1:**
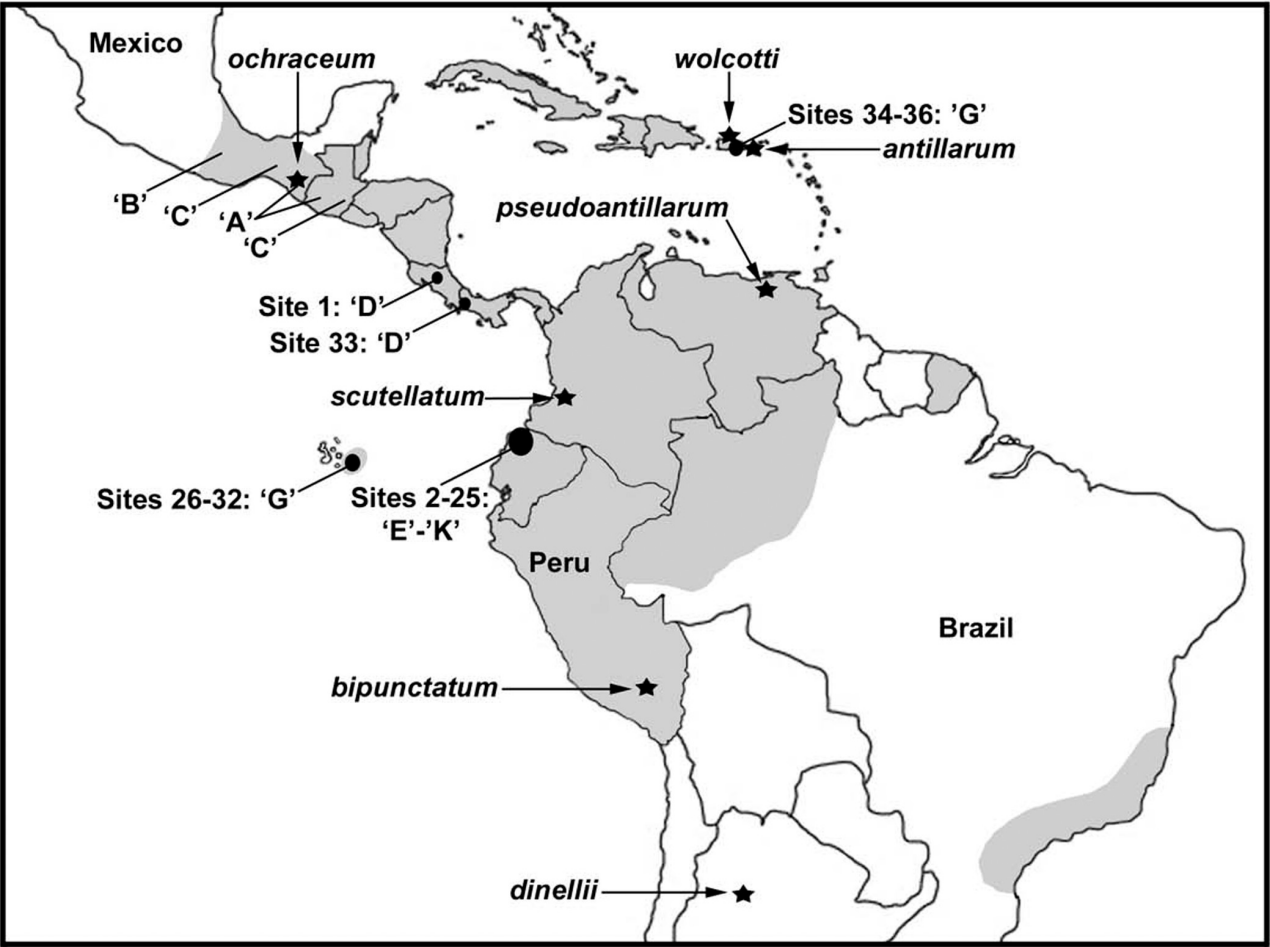
General distribution of *S*. *ochraceum* s. l. (gray shading) and collection sites (solid circles). Arrows point to type localities (stars). Letters in single quotes represent cytoforms; locations for cytoforms A–C are from an earlier study [25].

To center our investigation, we collected *S*. *ochraceum* sensu lato from 7 stream sites on San Cristobal Island in the Galapagos Archipelago. Although we obtained non-Galapagos samples from a wide geographic area (Fig 1), we concentrated our mainland search in what we considered the most likely source area for the Galapagos population—northwestern Ecuador where *S*. *ochraceum* had previously been reported from Esmeraldas Province [21]. Of 46 sites positive for black flies in mainland Ecuador, 26 had *S*. *ochraceum* s. l., with 24 sites having larvae large enough to analyze chromosomally. We also attempted to collect samples near type localities of taxa once regarded as species but now held in synonymy with *S*. *ochraceum*. The following 4 Puerto Rican samples were taken near the type locality (Cayey) of *S*. *wolcotti* Fox: one sample within 9 km east of the type locality and three samples about 45 km to the northeast. Although we sampled all running water on St. Croix, United States Virgin Islands, the type locality of *S*. *antillarum*, from 7 to 9 June 2016 and again from 14 to 16 March 2017, our efforts did not reveal black flies. We did not have access to the type localities of *S*. *bipunctatum* (‘Rio Charape’, Peru), *S*. *pseudoantillarum* (Monagas State, Venezuela), or *S*. *scutellatum* (Valle del Cauca Department, Colombia), all currently treated as synonyms of *S*. *ochraceum* (Fig 1). We also examined samples from Costa Rica and Panama.

### Collection and curation of specimens

Larvae were collected from all available substrates (e.g., fallen leaves, rocks, and trailing grasses) at 37 stream sites in 4 countries (Table 1) and fixed in 1:3 acetic ethanol (Carnoy’s solution) for chromosomal analysis; fixative was replaced immediately after collection and twice again over the next 12 h. Fixed samples were stored in the laboratory at 4°C until processing. Accurate identification of simuliids, even to species complex, typically requires multiple life stages, as well as chromosomes. Thus, we used forceps to collect pupae (when available), placed them in petri dishes lined with moist filter paper, held them at ambient temperature until adults emerged, and then fixed the adults in 95% ethanol after at least 12 hours of cuticular tanning. Adults were subsequently dehydrated through an ethanol series, chemically dried with hexamethyldisalazane [36], mounted with a minuten pin through the pleuron, and associated with the pupal exuviae in a microvial of glycerin. Larval carcasses, associated life stages, and photographic negatives of chromosomes were deposited in the Clemson University Arthropod Collection, Clemson, SC.

**Table 1:** Collections of larvae and associated life stages of *Simulium ochraceum* complex, 1999–2018.

Site No.	Location	Latitude	Longitude	Elevation (m asl^a^)	Collection Date	Cytoform (*n*)
	**COSTA RICA**					
1	San José Province, Moravia, Zurqui de Moravia Creek	10°02’50" N	84°00’30" W	1586	9 Aug 2013	D (12)
	**ECUADOR, mainland**					
2	Esmeraldas Province, SW of Quinindé	00°18’09" N	79°29’26" W	95	22 May 2014	K (2)
3	Esmeraldas Province, Mache-Chindul Ecological Reserve	00°23’27" N	79°38’51" W	350	15 May 2014	G (6)
4	Esmeraldas Province	00°22’54" N	79°34’29" W	135	15 May 2014	E2 (3), G (30)
5	Esmeraldas Province, N of Coronel Carlos Concha Torres	00°44’58" N	79°41’23" W	95	16 May 2014	E2 (14), F (11)
6	Esmeraldas Province, tiny tributary of Canande River	00°27’27" N	79°09’30" W	143	17 May 2014	K (15)
7	Esmeraldas Province	00°25’55" N	79°10’31" W	169	17 May 2014	E1 (3)
8	Esmeraldas Province,	00°53’00" N	79°34’34" W	56	20 May 2014	E2 (2), G (3)
9	Esmeraldas Province	00°51’52" N	79°33’05" W	150	20 May 2014	E2 (2)
10	Esmeraldas/Carchi Province	00°51’45" N	78°27’11" W	568	21 May 2014	E3 (4), H (1), K (9)
11	Esmeraldas/Carchi Province	00°51’47" N	78°27’11" W	567	21 May 2014	E3 (8), H (1)
12	Esmeraldas/Carchi Province	00°51’47" N	78°27’31" W	688	21 May 2014	E3 (11), H (1)
13	Esmeraldas/Carchi Province	00°51’37" N	78°27’37" W	719	21 May 2014	E3 (7), H (1)
14	Esmeraldas Province	00°51’06" N	78°27’59" W	1004	21 May 2014	E3 (9)
15	Esmeraldas Province	00°50’19" N	78°28’49" W	1157	21 May 2014	E3 (7), I (1)
16	Esmeraldas Province	00°48’35" N	78°29’39" W	1325	22 May 2014	E3 (8)
17	Esmeraldas Province	00°48’32" N	78°29’40" W	1341	22 May 2014	E3 (2), J (2)
18	Esmeraldas Province	00°48’48" N	78°29’27" W	1279	22 May 2014	E3 (11)
19	Esmeraldas Province	00°48’48" N	78°29’23" W	1266	22 May 2014	E3 (11)
20	Esmeraldas Province	00°48’50" N	78°29’19" W	1267	22 May 2014	K (2)
21	Esmeraldas Province	00°49’50" N	78°28’53" W	1171	22 May 2014	E3 (8), H (20)
22	Esmeraldas Province	00°49’50" N	78°28’54" W	1174	22 May 2014	K (34)
23	Esmeraldas Province	00°50’09" N	78°28’59" W	1160	22 May 2014	E3 (1), H (7)
24	Esmeraldas Province, Chuchuvi River, Hwy. 10	00°52’58" N	78°30’53" W	724	22 May 2014	H (2)
25	Pichincha Province	00°16’22" N	79°08’25" W	331	18 May 2014	E1 (4), K (6)
	**ECUADOR, Galapagos**					
26	San Cristobal, La Soledad	00°53’36" S	89°32’19" W	408	21 May 2014	G (23)
27	San Cristobal, El Chino (district), El Progreso (parish)	00°54’49" S	89°27’29" W	195	21 May 2014	G (21)
28	San Cristobal, near Jatun Sacha Research Facility	00°55’38" S	89°29’55" W	195	22 May 2014	G (23)
29	San Cristobal, Tres Palos (district), Progreso (parish)	00°55’45" S	89°30’06" W	178	22 May 2014	G (34)
30	San Cristobal, Cerro Gato	00°55’23" S	89°28’31" W	178	22 May 2014	G (23)
31	San Cristobal, La Policia (stream), Los Arroyos (district)	00°55’18" S	89°31’07" W	315	22 May 2014	G (33)
32	San Cristobal, Cerro Azul	00°55’07" S	89°32’36" W	308	23 May 2014	G (12)
	**PANAMA**					
33	Chiriquí Province, near Coffee Duran factory, tributary Rio Colorado	08°49’55"N	82°43’02"W	1215	24 June 1999	D (5)
	**PUERTO RICO**					
34	Luquillo Municipality, Rio Pitahaya	18°19’12" N	65°43’48" W	112	16 April 2016	G (12)
35	Rio Grande Municipality, Rio Espíritu Santo	18°21’36" N	65°48’36" W	43	16 April 2016	G (8)
36	Rio Grande Municipality, Rio Mameyes	18°18’36" N	65°46’12" W	406	23 April 2016	G (23)
37	Cayey Municipality, Hwy. 184	18°07’41" N	66°04’14" W	530	16 July 2018	G (2)

Note: Collections were made by P. H. Adler and J. W. McCreadie (mainland Ecuador), A. Borkent (Costa Rica), J. W. McCreadie (Galapagos Islands), M. McCormick (Puerto Rico, Cayey Municipality), and W. K. Reeves (Panama and Puerto Rico).

^a^ asl = above sea level.

### Cytogenetic analysis

Given the taxonomic confusion involving the *S*. *ochraceum* complex, we cast a wide net and analyzed specimens conforming to the morphological description of *S*. *ochraceum* s. l. [22]: larvae with a postgenal cleft nearly reaching the hypostoma and widest at midlength and with associated pupae with 8 gill filaments. To test for structural correlates of potential chromosomal entities and for sexual dimorphism in morphology, we presorted all larvae according to selected characters, viz., head-spot pattern, body color, and pigmentation pattern. Larvae were then prepared chromosomally using the Feulgen-staining procedure [37]. Polytene chromosomes from larval silk glands and one gonad for sex determination were slide mounted in 50% acetic acid and flattened with thumb pressure [1]. Representative sequences from high-quality nuclei were photographed under oil immersion on an Olympus BH-2 compound microscope with photographic film or on an Olympus BX40 compound microscope with a Jenoptik ProgRes® SpeedXT Core 5 digital camera. Photographic negatives were scanned and imported, or digital images were directly imported into Adobe® PhotoShop® Elements 8 to prepare chromosomal maps.

Our conventions for numbering inversions and chromosome sections followed those previously established for the *S*. *ochraceum* complex [25]. New inversions were numbered to begin with the last number previously assigned in each arm of the earlier chromosomal study [25]; the last numbered inversions in that study were IS-15, IL-13, IIS-8, IIL-7, IIIS (none), and IIIL-17. Heterochromatic blocks (hc), heavy bands (hyb), and supernumerary band insertions (in) were labeled for the section in which they occurred (e.g., IS hc36).

We compared the sequences of our polytene chromosome preparations with the standard sequence for the *Simulium ochraceum* complex previously established [25], which was based on the sequence represented in two cytoforms (A and B) of the three that were originally mapped. We used arrows or brackets on our photographic maps to indicate all rearrangements discovered in our analyses. Fixed inversions are underlined on the maps and italicized in the text. If the same inversion is fixed in one cytoform but polymorphic in another, it is italicized only when referring to the cytoform in which it is fixed. Sex chromosomes were identified by rearrangements, such as inversions, linked to sex on any of the three chromosomes (I, II, or III) in either the short (S) or long (L) arm. If no rearrangements were linked to sex, the sex chromosomes were considered microscopically (cytologically) undifferentiated (X_0_Y_0_).

### Terminology

We use “cytoform” as a general term for a chromosomally distinct segregate that can be recognized at an individual or a population level, with no implication as to whether it is capable of interbreeding (cytotype) or reproductively isolated (cytospecies) [1]. We designated each cytoform with a letter, following precedent [25] for the *S*. *ochraceum* complex. For closely related cytoforms, we appended a number to the letter (i.e., E1, E2, and E3), following previous convention [38].

We adopted a Mayrian [39] view of biological species, and evaluated the degree of reproductive isolation among cytoforms, based predominantly on chromosomal criteria, particularly absence of hybrids in sympatry [40], as well as ecological and geographical information. We then used available associated life stages to tie cytoforms to existing formal species names when possible.

A large vocabulary has built up around invasion biology, and attempts to standardize the terminology have had little success [5,41]. We use the familiar and universally understood term “native” to mean indigenous. Accordingly, native species would not necessarily be restricted to a particular location, such as the Galapagos Islands. Although the term “endemic” has often been used to refer to a species confined to the particular area under discussion, it is considered inappropriate in referring to distributions of species, and the term “precinctive” has been recommended as a replacement [42]. Thus, a native species found nowhere other than the Galapagos Islands would be precinctive to the Galapagos. We use “adventive” in the sense of previous workers [42] to mean non-native. Adventive species can arrive in a previously unoccupied area either through deliberate means (introduced species) or of their own volition, even if inadvertently via human agency (immigrant species) [42,43]. The arrival time of immigrant species is often unknown, blurring the distinction between adventive and native species. We use the term “invasive species” in accordance with established usage [41]: non-native (i.e., adventive) species that become established and cause adverse effects in their new environment.

## Results

### Chromosomal generalities

**Fig 2:**
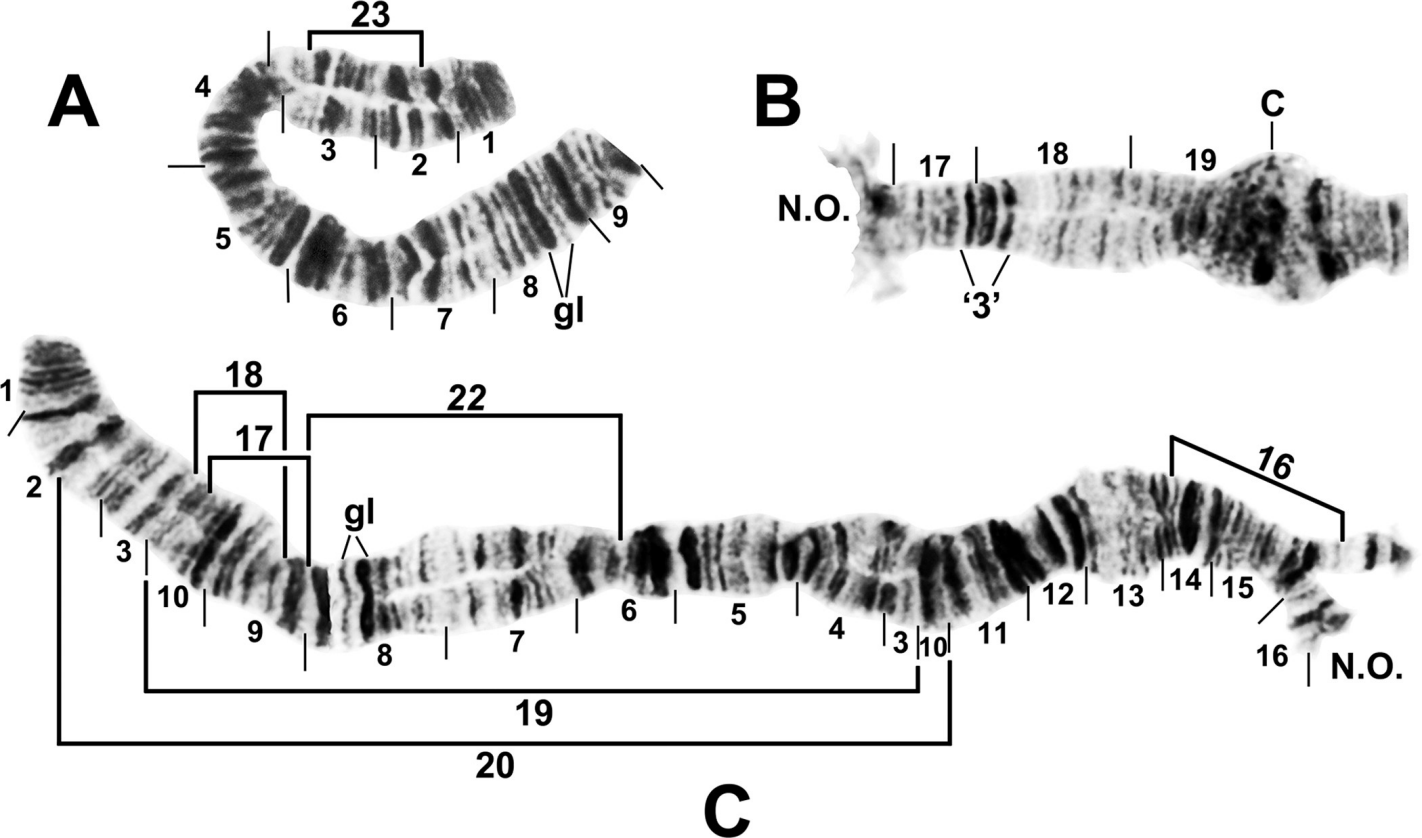
IS chromosome arm of *Simulium ochraceum* s. l. (**A**) Cytoform E1 (male larva, site 7), showing IS-23 heterozygote with inverted sequence in the top homologue. (**B** and **C**) Cytoform D (male larva, site 1), showing the nearly fixed IS-19 sequence; other autosomal inversions of *Simulium ochraceum* s. l. are indicated by brackets. C = centromere, gl = glazed, N.O. = nucleolar organizer, ‘3’ = 3 heavy.

**Fig 3:**
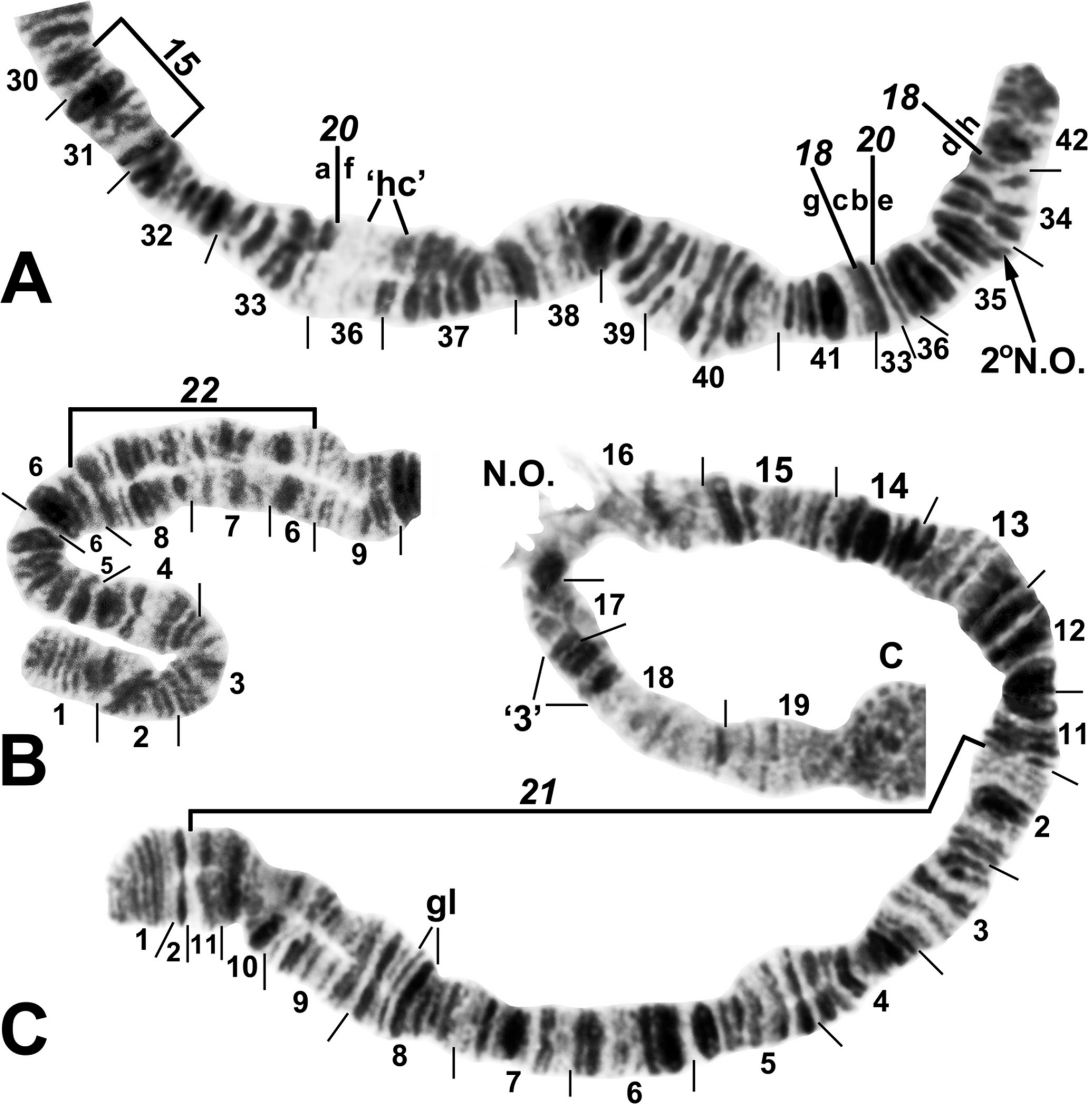
Chromosome I of *Simulium ochraceum* s. l. (**A**) Cytoform H (female larva, site 11), showing the *IL-18*,*20* sequence; basal sections of arm not shown. Letters a–h, when alphabetically ordered, produce the standard sequence for *S*. *ochraceum* s. l. Breakpoints of IL-15 are indicated by bracket. Arrow indicates location of secondary nucleolar organizer (2°N.O.) in cytoform E1; ‘hc’ indicates location of heterochromatic block in cytoform E3. (**B**) Cytoform H (female larva, site 12), showing *IS-22* sequence. (**C**) IS-21 sequence of cytoform J (female larva, site 17). C = centromere, gl = glazed, N.O. = nucleolar organizer.

Among 500 fully analyzed larvae of *S*. *ochraceum* s. l., we found 13 distinct chromosomal segregates (Table 2); three additional larvae (0.6%) prepared for chromosomal examination could not be fully analyzed and were not used in any analyses. All entities were chromosomally defined by nucleolar expression in section 16 of IS (Figs 2 and 3), tightly paired homologues, and expanded centromere regions. These chromosomal features and the standard band sequence of the complement provided a second filter to ensure that morphologically similar larvae that did not belong to the *S*. *ochraceum* complex were excluded from analyses. Thus, we could exclude larvae of the *S*. *lutzianum* complex, which have a postgenal cleft and head-spot pattern similar to those of *S*. *ochraceum* s. l. [21] and were present at 15 of our Esmeraldas sampling sites. Identification of these larvae was confirmed by comparison with relevant chromosome maps [44] that revealed three cytoforms (A1, A2, and B) of *S*. *lutzianum*, a member of the *S*. *romanai* species group [22]. The location of the nucleolar organizer in the extreme base of IS, within the expanded centromere region, provides a quick diagnostic aid for the *S*. *lutzianum* complex.

**Table 2:** Diagnostic inversions, sex chromosomes, formal names, and distributions of cytoforms of the *Simulium ochraceum* complex.

Cytoform^a^	Diagnostic inversions^b^	Sex chromosomes^c^	Formal name	Geographic distribution	Elevation (m asl)
A	[standard sequence]	X_0_X_0_, X_0_X_1_, X_0_Y_0_, X_0_Y_1_, X_0_Y_2_, X_0_Y_3_, X_0_Y_4_, X_0_Y_5_	*Simulium ochraceum* sensu stricto	Guatemala, Mexico	900–1700
B	[standard sequence]	X_1_X_1_, X_1_X_2_, X_2_X_2_, X_1_Y_0_, X_1_Y_1_, X_2_Y_0_, X_2_Y_1_	?	Mexico	1600
C	*IIS-7*, *IIIL-12*a,*13a*,*14a*,*15a*	X_0_X_0_, X_0_Y_1_	?	Guatemala, Mexico	600
D	(IS-19), *IIS-7*,*8*, (IIS-13), *IIL-7*, (IIIS-2), *IIIL-12*a,*13a*,*14a*,*15a*	X_1_X_1_, X_1_X_3_, X_2_X_2_, X_2_X_3_, X_3_X_3_, X_3_X_4_, X_3_X_5_, X_1_Y_0_, X_2_Y_2_, X_3_Y_1_, X_3_Y_3_	?	Costa Rica, Panama	1215–1586
E1	*IIS-15*, *IIIS-1*, *IIIL-20*,*21*, (IIIL-23)	X_0_X_0_, X_0_Y_0_	?	Ecuador (mainland)	169–331
E2	*IIS-15*, *IIIS-1*, *IIIL-20*,*21*,*23*, (IIIL-24)	X_0_X_0_, X_0_Y_0_	?	Ecuador (mainland)	56–150
E3	*IIS-15*, *IIIS-1*, *IIIL-20*, (IIIL-21), (IIIL-22), (IIIL-23)	X_0_X_0_, X_0_Y_0_	?	Ecuador (mainland)	567–1341
F	*IIS-16*,*17*, *IIIL-14a*	X_0_X_0_, X_0_Y_0_	?	Ecuador (mainland)	95
G	*IIS-18*, *IIL-14*,*15*,*16*,*17*,*18*, *IIIS-1*, *IIIL-14a*,*27*,*28*,*29*,*30*	X_0_X_0_, X_0_Y_1_	*Simulium antillarum* (synonym: *S*. *wolcotti*)	Ecuador (mainland and Galapagos Islands), Puerto Rico	43–530
H	*IS-22*, *IL-17*,*18*,*20*, *IIS-9*, *IIL-7*, (IIIS-1), *IIIL-14a*	X_1_X_3_, X_3_X_3_, X_2_Y_3_, X_3_Y_1_, X_3_Y_2_, X_3_Y_4_	?	Ecuador (mainland)	567–1171
I	*IS-22*, *IL-17*,*18*, *IIS-11*, *IIIL-14a*,*36*,*37*	?	?	Ecuador (mainland)	1157
J	*IS-21*, *IL-17*, *IIL-7*, *IIIS-1*,*5*,*6*, *IIIL-38*,*39*	X_0_X_0_, X_0_Y_0_	?	Ecuador (mainland)	1341
K	*IL-17*,*18*, *IIS-19*, *IIIL-14a*	X_0_X_0_, X_0_Y_0_	*Simulium dinellii*	Ecuador (mainland)	95–1267

^a^ Data for cytoforms A, B, and C are from an earlier chromosomal study [25].

^b^ Fixed inversions are italicized; polymorphic inversions with overall frequencies > 0.50 for a cytoform are parenthetical.

^c^ Details of differentiated sex chromosomes are given under respective cytoform treatments.

We discovered 88 rearrangements among the 13 cytoforms, of which 86.4% were paracentric inversions (S1 Table). No pericentric inversions were represented. The remaining 13.6% of the rearrangements included supernumerary (B) chromosomes, differential centromere bands, heterobands, heterochromatic blocks, supernumerary band insertions, differential nucleolar expression, secondary nucleolar organizer expression, and a translocation. The mean number of heterozygous autosomal inversions per larva varied from 0.00 in cytoforms F and J to 2.00 in cytoforms D and I. Combined with rearrangements discovered in a previous study [25], the *S*. *ochraceum* complex now includes 142 rearrangements distributed throughout the complement as follows: IS (24), IL (27), IIS (20), IIL (19), IIIS (7), IIIL (41), centromeres (2 forms), and B chromosomes (2 types). Among the 500 larvae, we found no hybrids.

### Cytoform D

**Fig 4:**
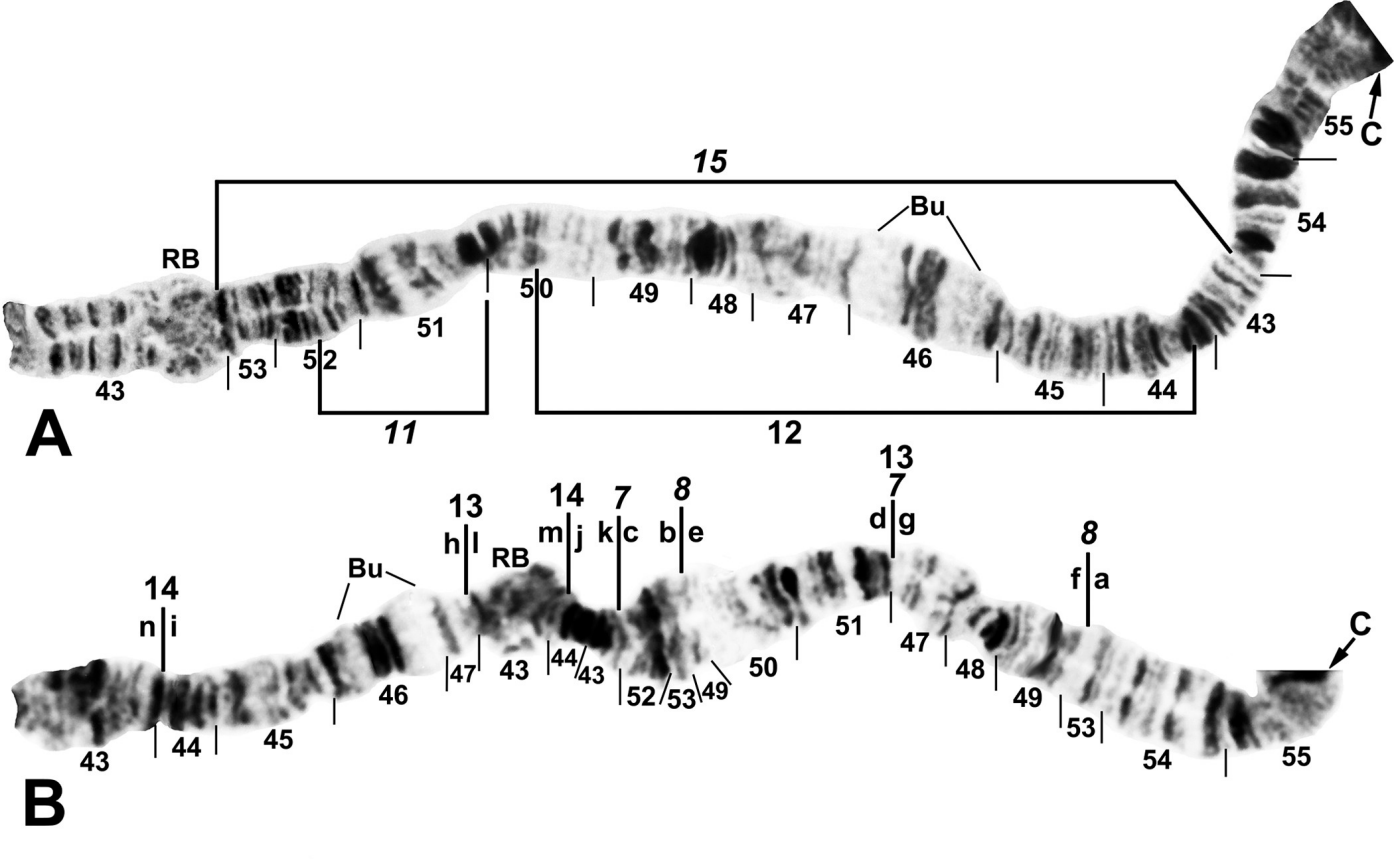
IIS arm of *Simulium ochraceum* s. l. (**A**) Cytoform E2 (female larva, site 4), showing the *IIS-15* sequence. Diagnostic inversions *IIS-11* and IIS-12 of cytoform I are indicated by brackets. (**B**) Cytoform D (female larva, site 1), showing the common *IIS-7*,*8*,13,14 sequence. Alphabetizing the letters a–n will produce the standard sequence for *S*. *ochraceum* s. l. Bu = bulge, C = centromere, RB = ring of Balbiani.

**Fig 5:**
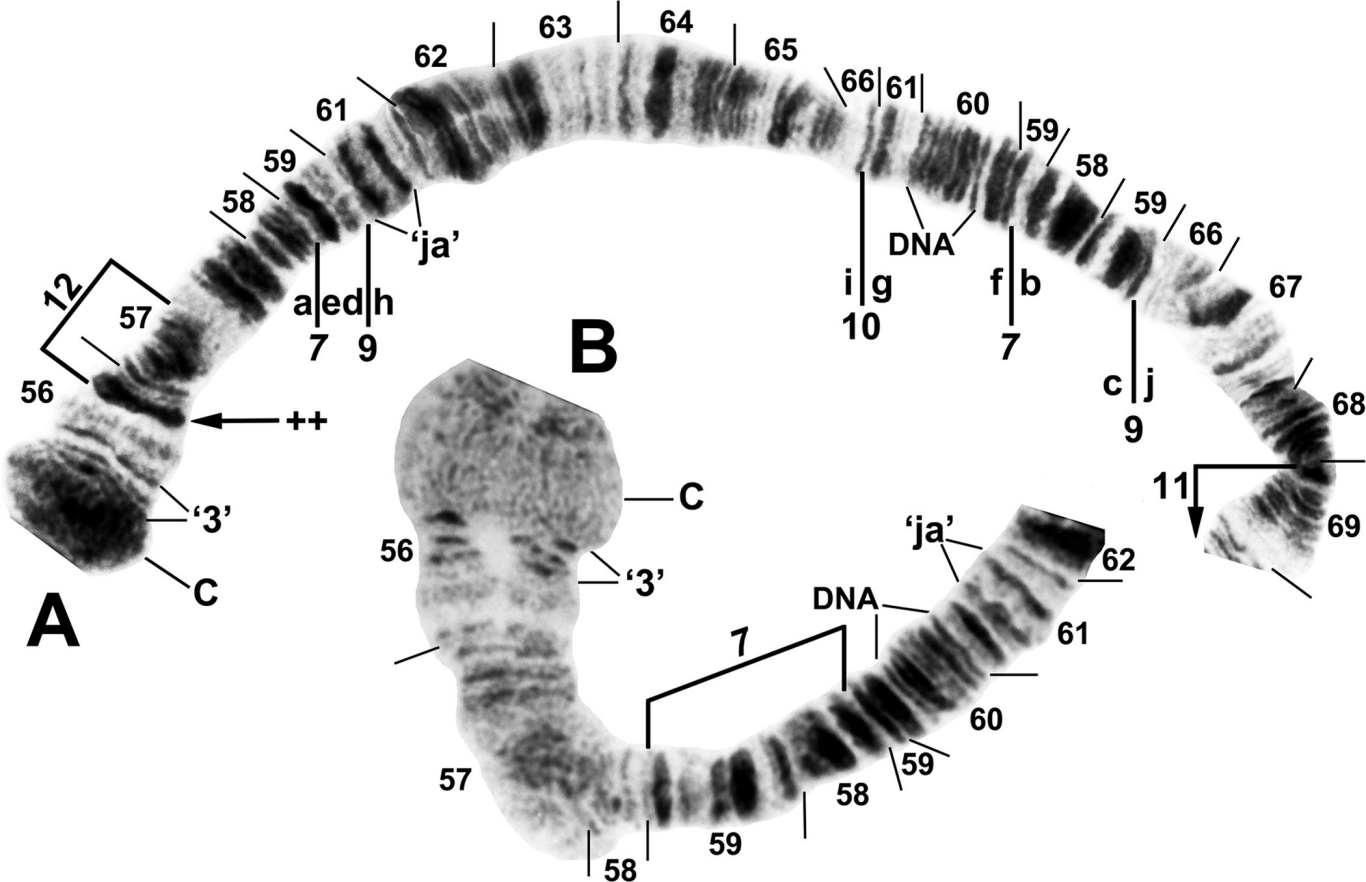
IIL arm of *Simulium ochraceum* s. l. (female larvae). (**A**) Cytoform D (Panama), showing the common *IIL-7*,9,10 X-chromosome sequence; terminal sections of arm not shown. Alphabetizing the letters a–j will produce the standard sequence for *S*. *ochraceum* s. l. Breakpoints of IIL-12 and the proximal breakpoint of IIL-11 are indicated by brackets. C = centromere, DNA = DNA puff, ‘ja’ = jagged, ‘3’ = 3 sharp, ++ = homozygous expression of heavy band (hyb). (**B**) Cytoform H (site 11); base of IIL showing the *IIL-7* sequence.

**Fig 6:**
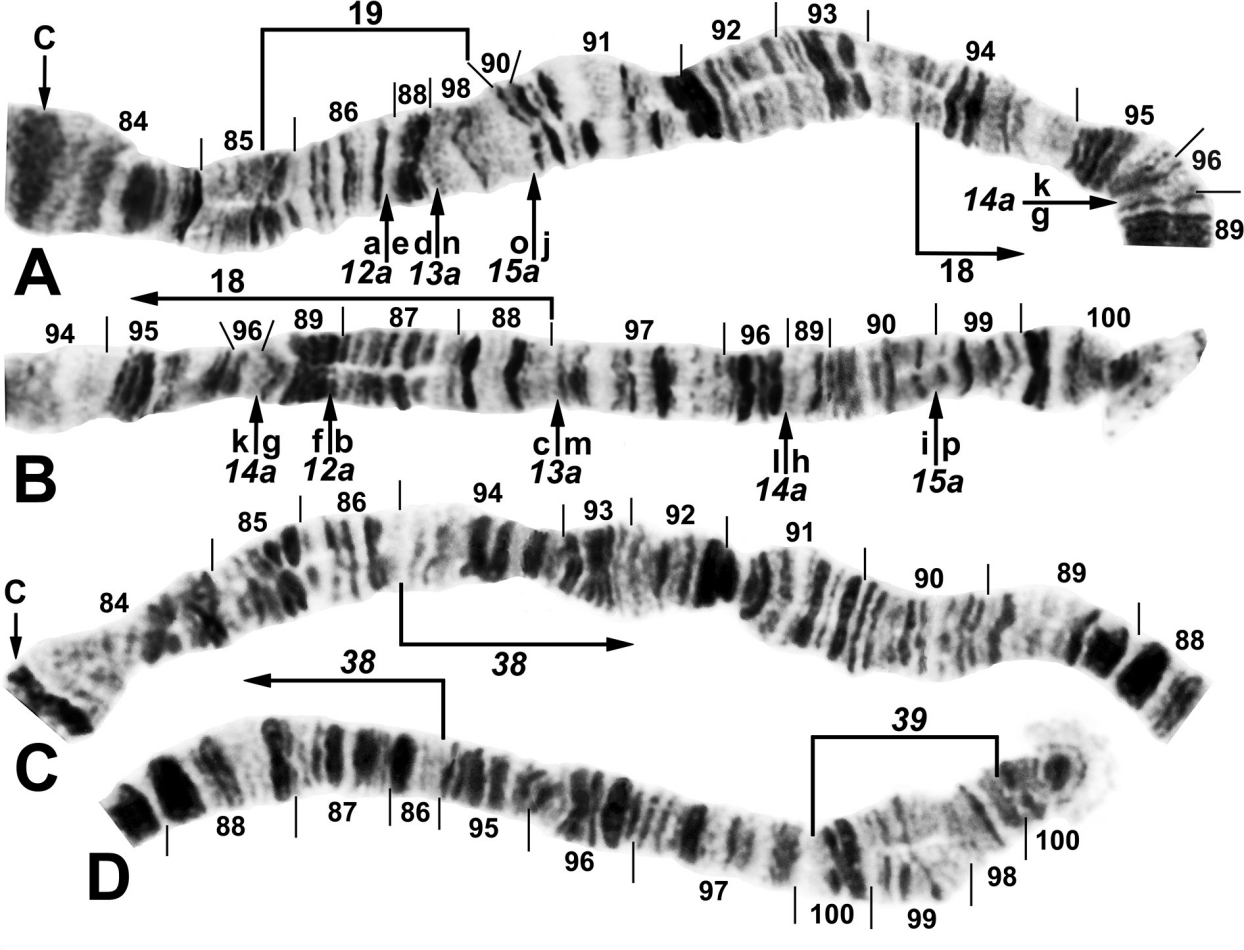
IIIL arm of *Simulium ochraceum* s. l. (**A** and **B**) Cytoform D (male larva, site 1), showing the *IIIL-12a*,*13a*,*14a*,*15a* sequence. Letters a–p, when alphabetically ordered, produce the standard sequence for *S*. *ochraceum* s. l. Limits of autosomal polymorphisms IIIL-18 and IIIL-19 are indicated by brackets. (**C** and **D**) Cytoform J (female larva, site 17), showing the *IIIL-38*,*39* sequence. C = centromere.

**Fig 7:**
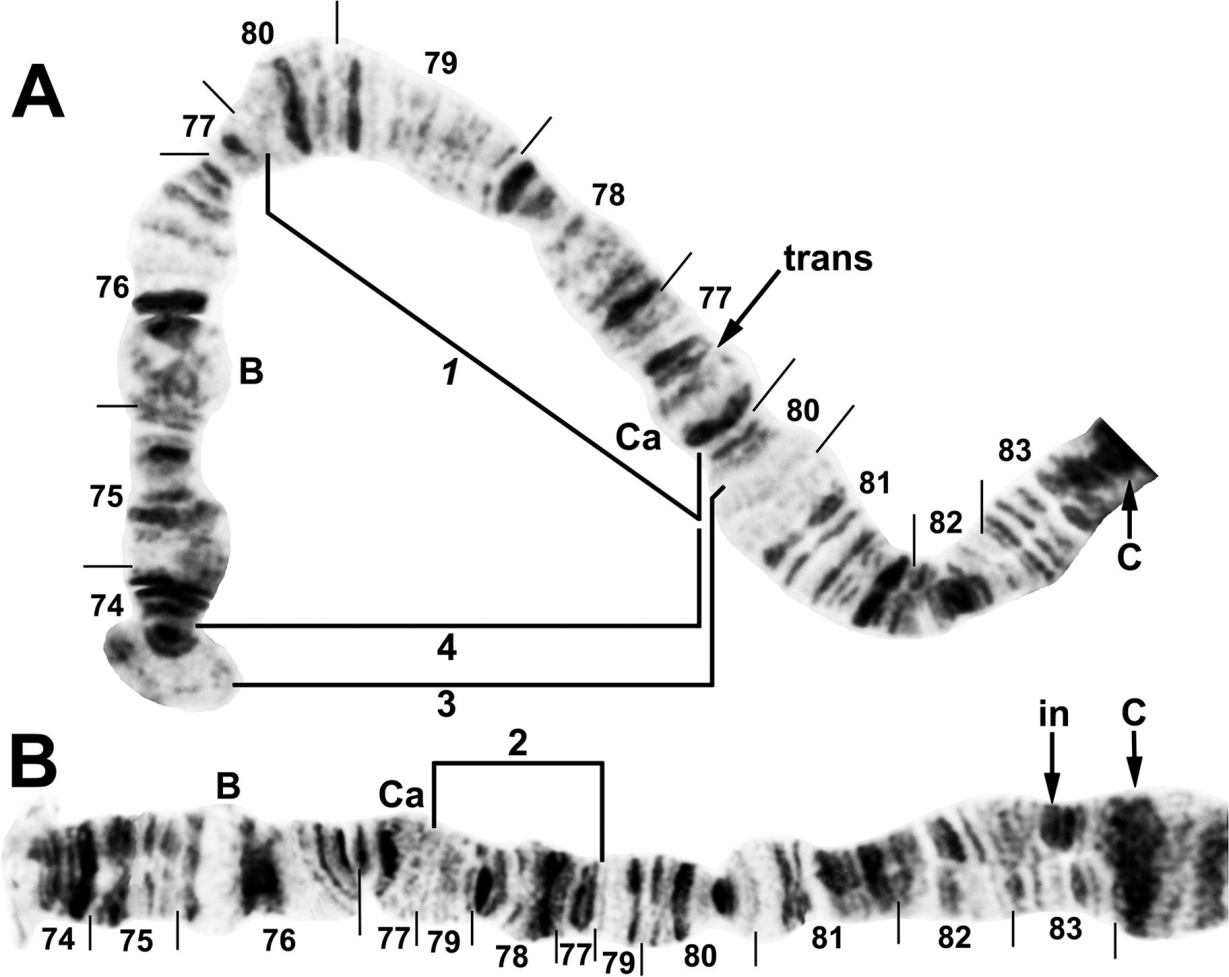
IIIS arm of *Simulium ochraceum* s. l. (**A**) Cytoform E2 (female larva, site 4), showing the *IIIS-1* sequence and brackets indicating limits of IIIS-3 and IIIS-4 of cytoform E3. (**B**) Cytoform D (male larva, site 1), showing the nearly fixed IIIS-2 sequence and heterozygous band insertion (in) in section 83. B = blister, C = centromere, Ca = capsule, trans = breakpoint of Galapagos translocation polymorphism.

**Fig 8:**
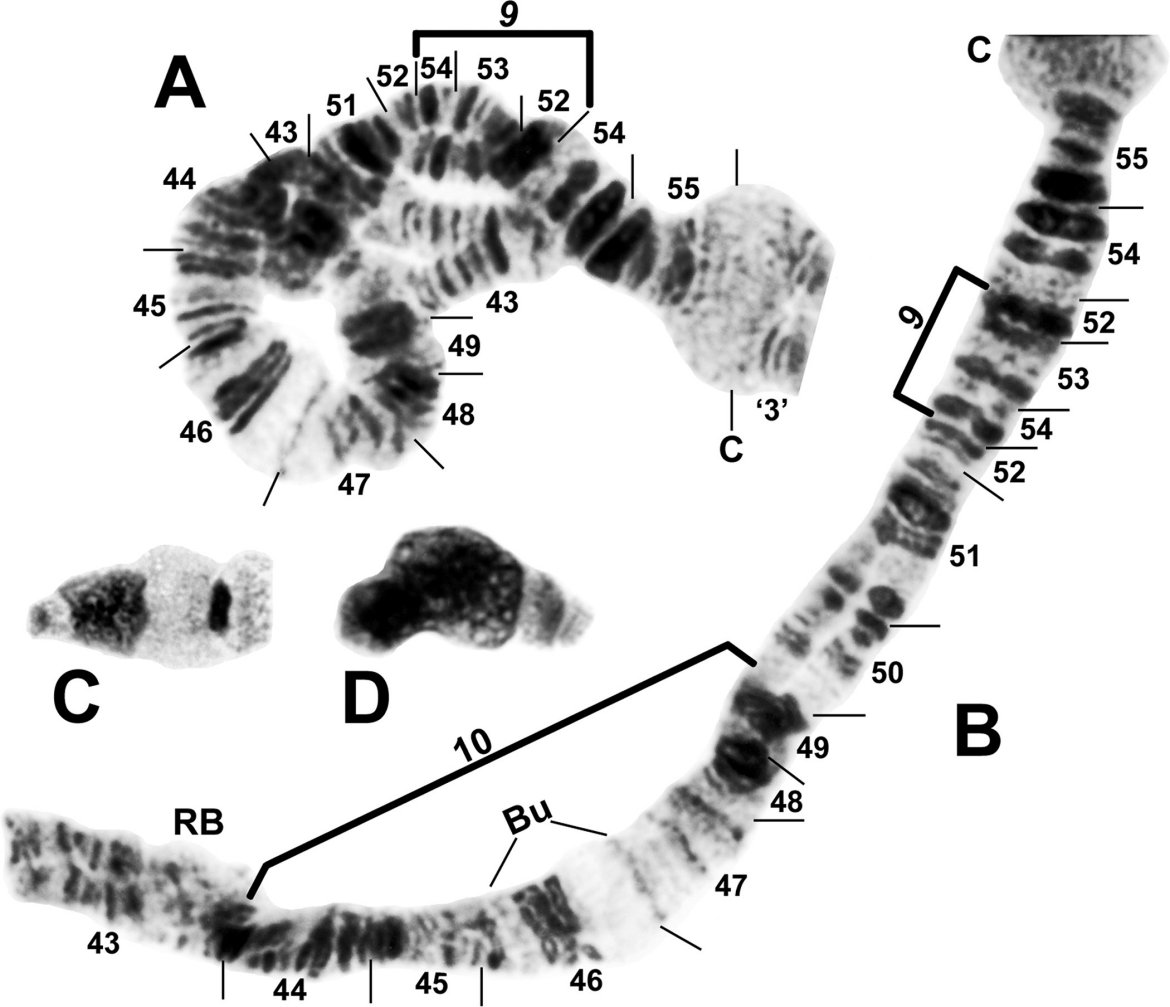
IIS arm and B chromosomes of *Simulium ochraceum* s. l. (**A** and **B)** Cytoform H (female larvae, site 11). Bu = bulge, C = centromere, RB = ring of Balbiani. (A) *IIS-9* sequence with heterozygous IIS-10 configuration. (**B**) Diagnostic *IIS-9* sequence; bracket indicates limits of autosomal polymorphism IIS-10. (**C** and **D**) B chromosomes (male larvae). (**C**) Cytoform D (site 1). (**D**) Cytoform H (site 21).

This new segregate was collected at one site 1586 m above sea level (asl) in Costa Rica and at one site 1400 m asl in Panama 220 aerial km to the southeast. It differed from the standard banding sequence by 7 fixed inversions: *IIS-7*, *IIS-8*, *IIL-7*, *IIIL-12a*, *IIIL-13a*, *IIIL-14a*, and *IIIL-15a* (Figs 4–6). Cytoform D also had 2 nearly fixed inversions, IS-19 (Fig 2) and IIIS-2 (Fig 7) with frequencies > 0.95/site; 2 high-frequency inversions (IIS-13 and IIS-14) on top of *IIS-7*,*8* (Fig 4); 10 additional polymorphisms (of which two were shared between Costa Rica and Panama); and B chromosomes (Fig 8) in the Costa Rican population (S1 Table). Centromere bands were variably distinct (Figs 2B and 6A). Ectopic pairing of centromere bands CII and CIII manifested in less than 10% of nuclei in one female.

**Fig 9:**
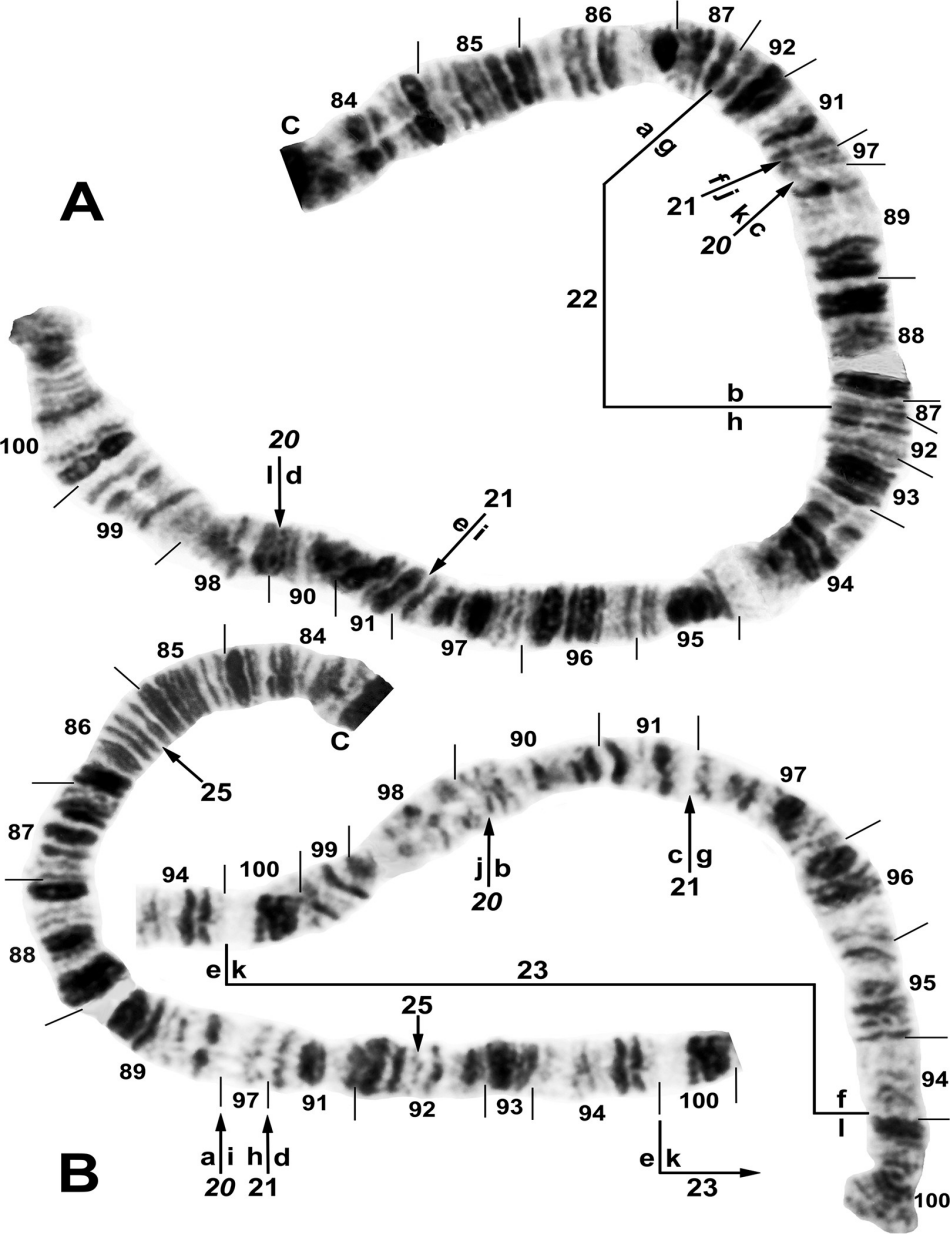
IIIL arm of *Simulium ochraceum* cytoform E3 (female larvae). (**A**) *IIIL-20*,21,22 sequence (site 15). Alphabetizing the letters a–l will produce the standard sequence for *S*. *ochraceum* s. l. (**B**) *IIIL-20*,21,23 sequence, with breakpoints of IIIL-25 indicated by arrows (site 11). Alphabetizing the letters a–l will produce the standard sequence for *S*. *ochraceum* s. l. C = centromere.

The fixed IIIL banding sequence of our larvae of cytoform D was identical to that of Guatemalan larvae of cytoform C presented in Fig 9A of an earlier study [25]. Although we agree that 4 fixed inversions are involved relative to the standard, our interpretation of breakpoints differs substantially, as we show in Fig 6A and 6B. Rather than assign new numbers to our inversions, we use the original numbers from the previous study [25] for the most similar inversions but append an ‘a’ to each; thus, *IIIL-12a*,*13a*,*14a*,*15a* indicates an amended sequence. Our revised solution to the inversion complex is critical to interpretation of relationships because *IIIL-14a* defines the basic IIIL sequence in 7 of the 13 total cytoforms.

IIL was the sex arm in cytoform D. In Costa Rica, 5 X sequences were recognized, where hyb was an enhanced (heavy) band in section 56 (Fig 5A): X_1_ = IIL-9,10; X_2_ = IIL-9,10+hyb; X_3_ = IIL-9,10,11+hyb; X_4_ = IIL-12+hyb; and X_5_ = IIL-9,10,12. Four Y sequences were found: Y_0_ = standard, Y_1_ = IIL-11+hyb, Y_2_ = IIL-12, and Y_3_ = IIL-12+hyb. Thus, the IIL-9,10 sequence provided the fundamental platform for the X chromosome, appearing in 18 of 19 X chromosomes, but none of the 5 Y chromosomes. The X and Y combined as follows: 1 X_1_X_3_, 1 X_2_X_2_, 1 X_2_X_3_, 2 X_3_X_3_, 1 X_3_X_4_, 1 X_3_X_5_, 1 X_1_Y_0_, 1 X_2_Y_2_, 2 X_3_Y_1_, and 1 X_3_Y_3_. In Panama, given the small sample size (*n* = 5 larvae), an interpretation of the sex chromosomes was not definitive. If the heavy band, which was expressed in all 10 homologues, is considered fixed, all 4 females were X_1_X_1_ and the 1 male was X_1_Y_0_.

We recognize cytoform D from Costa Rica and Panama as distinct from cytoform C previously recognized from Guatemala [25]. Although the two cytoforms share 5 fixed inversions (*IIS-7* and *IIIL-12a*,*13a*,*14a*,*15a*), plus similar B chromosomes (here considered homologous) and two additional inversions (IIS-8 and IIL-7) that are fixed in D but polymorphic in C, they differ in their sex chromosomes (IIL arm in D, IIIL arm in C) and in unique sets of autosomal polymorphisms.

### Cytoforms E1, E2, and E3

**Fig 10:**
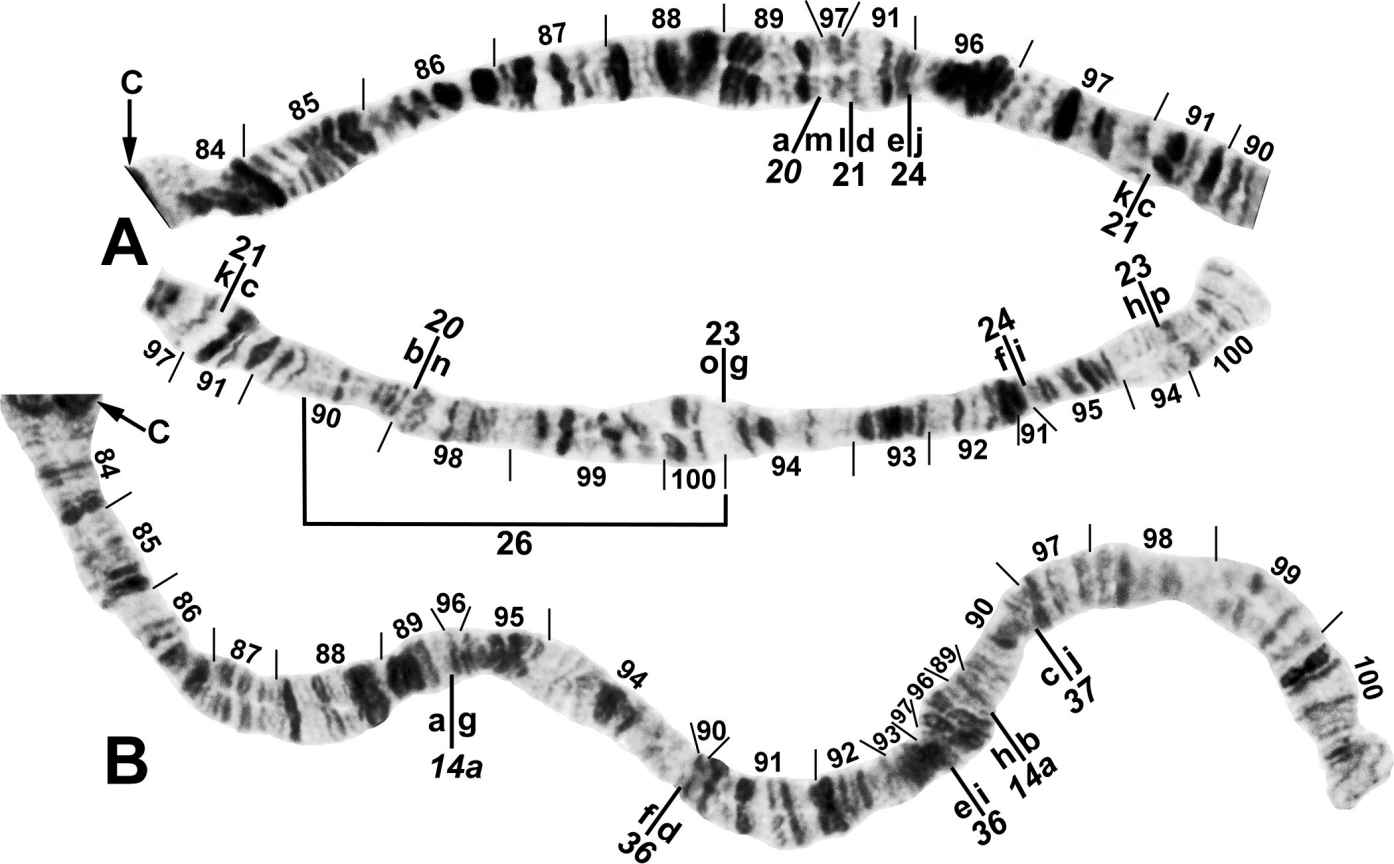
IIIL arm of *Simulium ochraceum* s. l. (**A**) Cytoform E2 (female larva, site 9), showing the *IIIL-20*,21,23,24 sequence, with breakpoints of IIIL-26 indicated by a bracket. Letters a–p, when alphabetically ordered, produce the standard sequence for *S*. *ochraceum* s. l. (**B**) Cytoform I (male larva, site 15), showing the *IIIL-14a*,*36*,*37* sequence. The second break for *IIIL-37* appears after *IIIL-36* is inverted to standard, creating the d/i junction. Alphabetizing the letters a–j will produce the standard sequence for *S*. *ochraceum* s. l. C = centromere.

**Fig 11:**
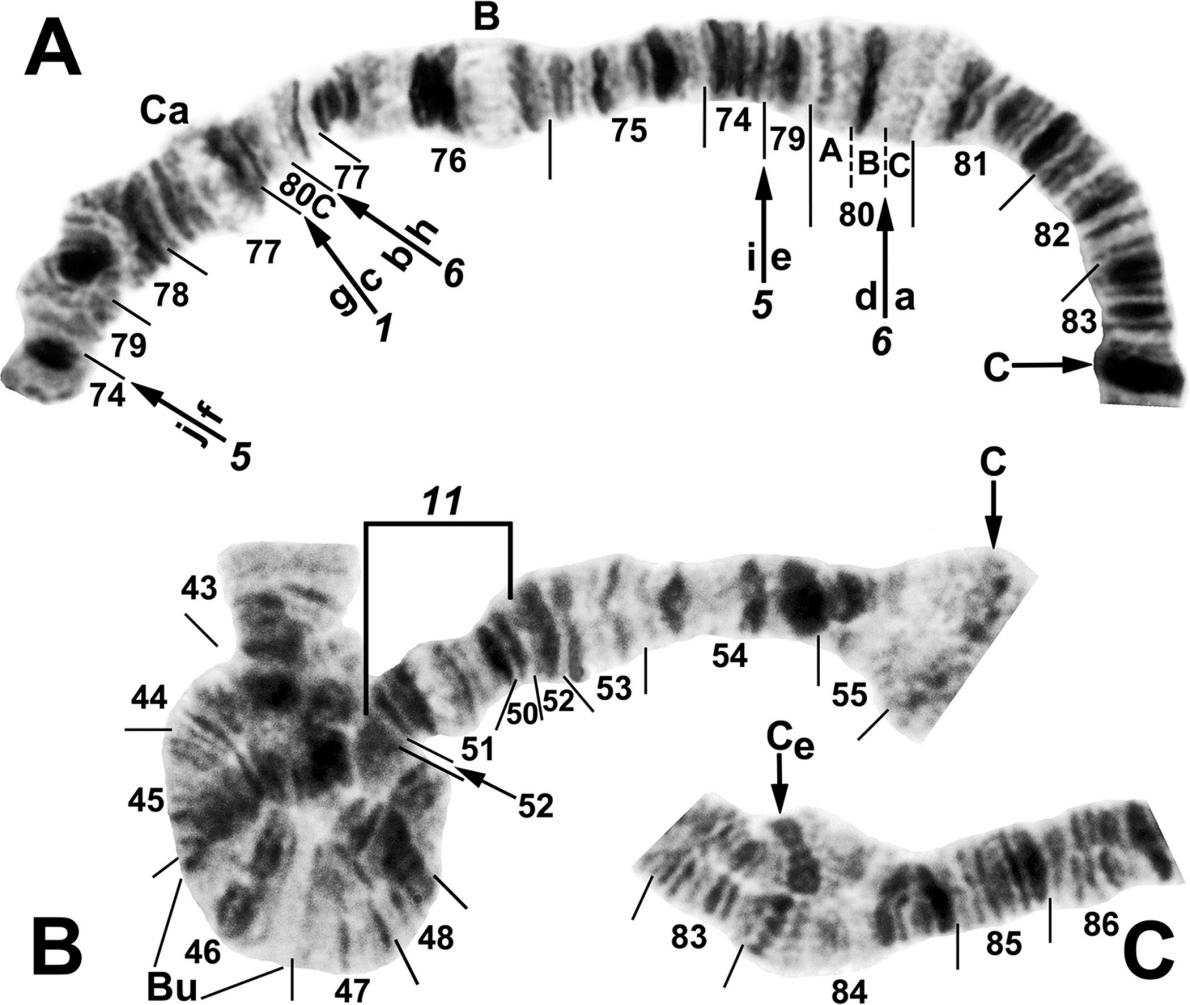
IIS and IIIS arms of *Simulium ochraceum* s. l. (**A**) Cytoform J (female larva, site 17), showing the *IIIS-1*,*5*,*6* sequence. *IIIS-1* and *IIIS-6* share a breakpoint; thus, assuming that *IIIS-1* was the first inversion that occurred relative to the standard, the second breakpoint for *IIIS-1* would be seen by inverting *IIIS-6* to standard. Letters a–j, when alphabetically ordered, produce the standard sequence for *S*. *ochraceum* s. l. B = blister, C = centromere, Ca = capsule. (**B**) Cytoform I (male larva, site 15), showing the homozygous *IIS-11* sequence with IIS-12 heterozygous. Bu = bulge. (**C)** Cytoform E1 (male larva, site 7), showing heterozygous configuration for the enhanced centromere III band (C_e_).

The E group of cytoforms was found in 18 of 46 streams positive for black flies in mainland Ecuador from 56 to 1335 m asl. The basic sequence of the E group differed chromosomally from the *S*. *ochraceum* standard sequence by 3 fixed inversions: *IIS-15* (Fig 4A), *IIIS-1* (Fig 7A), and *IIIL-20* (Figs 9A, 9B and 10A); in addition, IIIL-21 was nearly fixed (frequency across cytoforms = 0.96). Sex chromosomes were microscopically undifferentiated. The intensity of staining and degree of definition of centromeres varied among and within larvae. CII consistently stained darkly and was well defined, whereas CI and CIII were variable in expression, with rare (frequency = < 0.01) heterozygous expression (Fig 11C). Ectopic pairing of centromeres ranged from 10% to 85% of nuclei per larva.

Polymorphisms in the cytoform E group were infrequent overall (< 0.01), except for five in IIIL (S1 Table), which defined the three members of the E group, each corresponding to a particular geographic area in northwestern Ecuador. Cytoform E1, comprised of a small set of southern samples (sites 7 and 25), was fixed for *IIIL-21* and carried IIIL-23 in high frequency (0.93). Cytoform E2, consisting of western samples (sites 4, 5, 8, and 9), was fixed for *IIIL-21* and *IIIL-23* and had a high frequency (0.93) of IIIL-24 (Fig 10A). A northeastern set of samples (sites 10–19, 21, 23), representing cytoform E3, was polymorphic for IIIL-21 (0.93), IIIL-22 (0.54), IIIL-23 (0.53), and IIIL-25 (0.11) (S1 Table; Fig 9A and 9B).

Within the E group, only cytoform E3 had mermithid nematodes (S1 Table).

### Cytoform F

**Fig 12:**
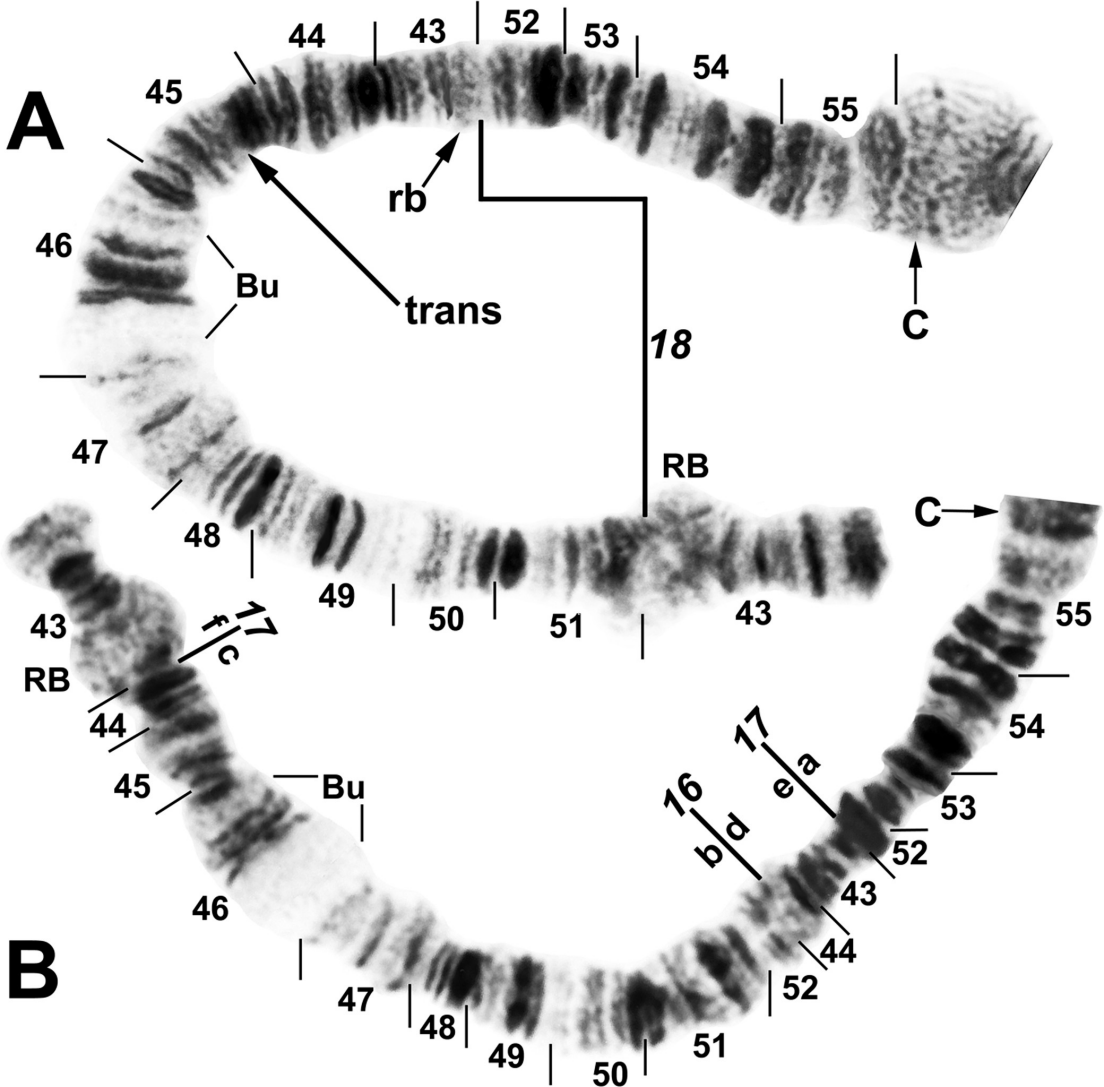
IIS arm of *Simulium ochraceum* s. l. (**A**) Cytoform G (male larva, site 34), showing the *IIS-18* sequence. (**B**) Cytoform F (male larva, site 5), showing the *IIS-16*,*17* sequence. Inverting *IIS-17* forms the second (coincident) breakpoint (a/c) for *IIS-16*. Alphabetizing the letters a–f will produce the standard sequence for *S*. *ochraceum* s. l. Bu = bulge, C = centromere, RB = ring of Balbiani, rb = possible cleaved edge of ring of Balbiani, trans = breakpoint of Galapagos translocation polymorphism.

**Fig 13:**
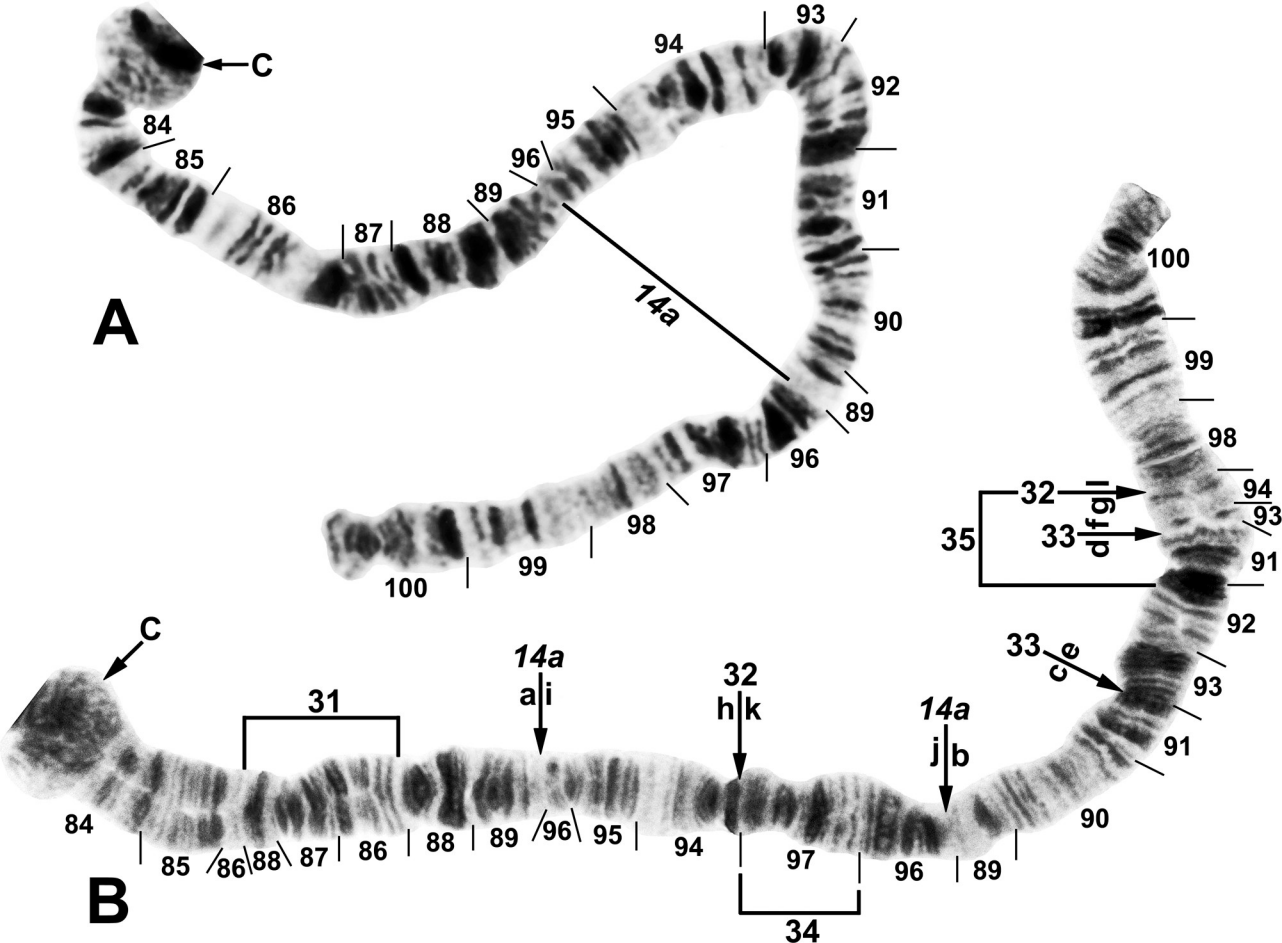
IIIL arm of *Simulium ochraceum* s. l. (**A**) Cytoform F (male larva, site 5), showing the *IIIL-14a* sequence. (**B**) Cytoform H (female larva, site 21), showing the typical X-chromosome *IIIL-14a*,31,32,33 sequence, with breakpoints of IIIL-34 and IIIL-35 indicated by brackets. Alphabetizing the letters a–l will produce the standard sequence for *S*. *ochraceum* s. l. C = centromere.

One small sample of 11 larvae from site 5 at 95 m asl in Ecuador’s Esmeraldas Province represented yet another distinct chromosomal entity. It was characterized by three fixed inversions—*IIS-16*, *IIS-17* (S1 Table, Fig 12B), and *IIIL-14a* (Fig 13A)—of which *IIIL-14a* was shared with 5 other cytoforms (plus cytoform C [25] (Hirai et al. 1994)). The unique IIS sequence had breakpoints (a/e and f/c, Fig 12B) deceptively similar to those for *IIS-7*. However, if our section numbering is correct for the d–e segment (Fig 12B), the most parsimonious derivation of IIS from standard is a two-step break involving cytoform-specific inversions *IIS-16* and *IIS-17*. The centromere bands were well defined. Sex chromosomes were microscopically undifferentiated, and polymorphisms were not found.

We collected cytoform F from a tiny forest stream (0.5 m wide) trickling over bedrock, where it was found with cytoform E2. Forty-five minutes of collecting by two of us yielded 65 larvae of the *S*. *ochraceum* complex, of which 25 were large enough for a band-by-band chromosomal analysis. No hybrids were present in our sample of 25 larvae, demonstrating that cytoforms E2 and F are reproductively isolated. The two cytoforms differed not only by 8 fixed inversions at site 5, but also by the degree of ectopic pairing: entirely absent in cytoform F, but present in 33–85% of nuclei per larva in cytoform E2 at the site, providing a useful diagnostic aid, even in small larvae with underpolytenized chromosomes.

### Cytoform G

**Fig 14:**
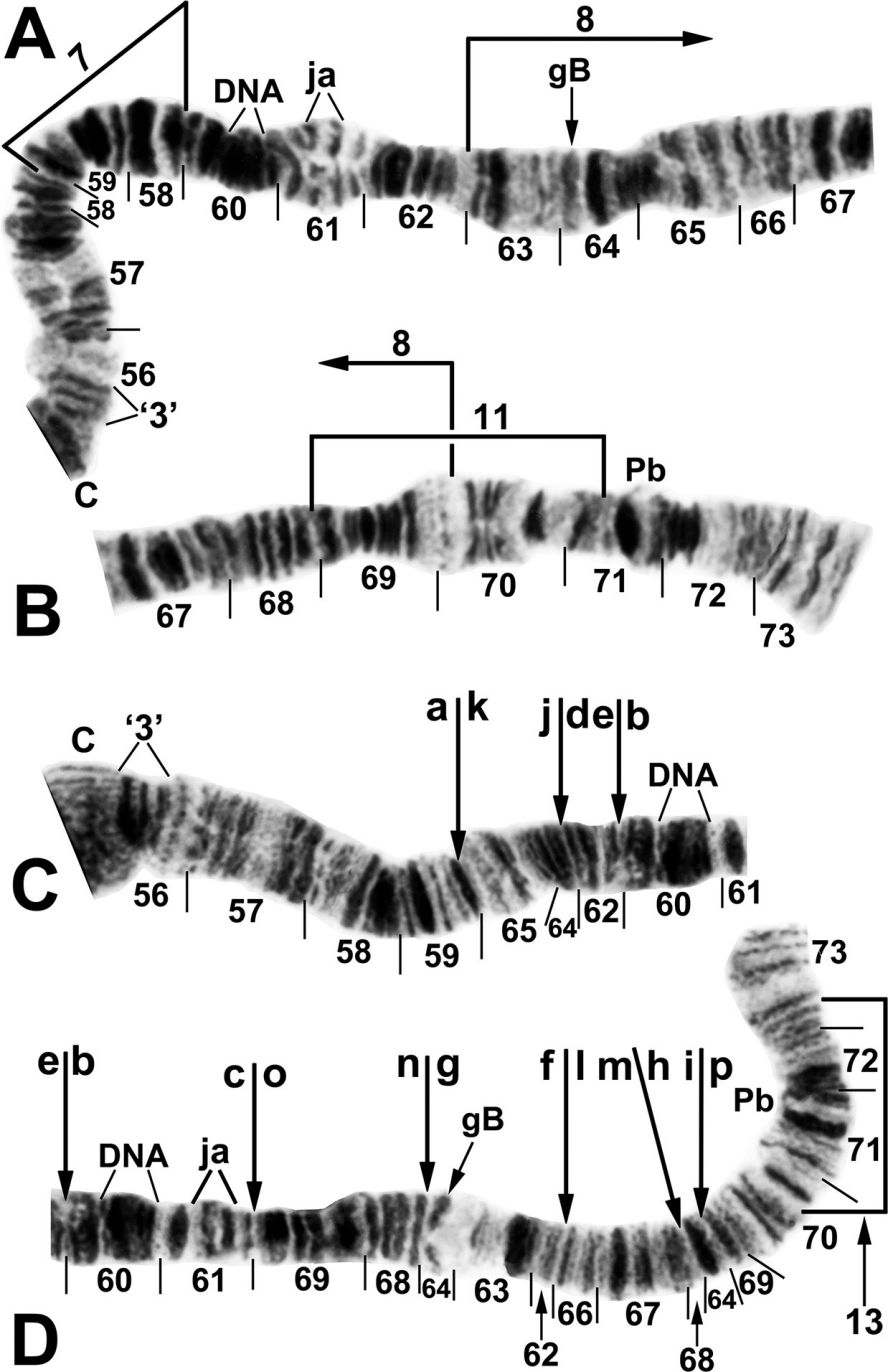
IIL arm of *Simulium ochraceum* s. l. (**A** and **B**) Cytoform J (female larva, site 17), showing *IIL-7* sequence. Breakpoints of autosomal inversion IIL-8 of cytoform H and sex-linked inversion IIL-11 of cytoform D are indicated by brackets. (**C** and **D**) Cytoform G, showing a complex of 5 fixed inversions (*IIL-14*,*15*,*16*,*17*,*18*), all of which are novel. Multiple breakpoint scenarios are possible; therefore, the breakpoints are not numbered on the map but are indicated with arrows. Alphabetizing the letters a–p will produce the standard sequence for *S*. *ochraceum* s. l. Breakpoints of autosomal inversion IIL-13 in the Galapagos Islands are indicated by a bracket. C = centromere, DNA = DNA puff, gB = gray band, Pb = parabalbiani, ‘3’ = 3 sharp.

**Fig 15:**
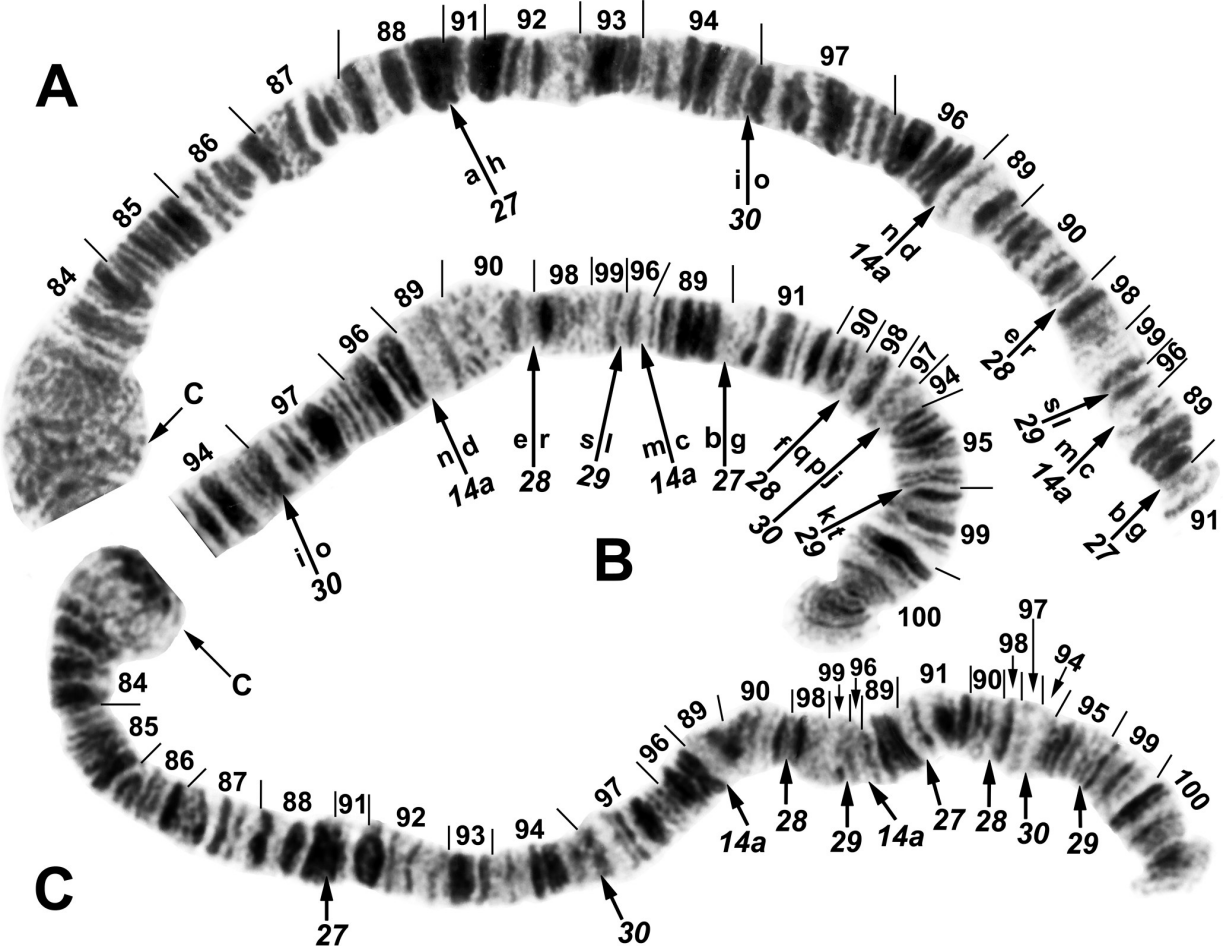
IIIL arm of *Simulium ochraceum* cytoform G, showing the *IIIL-14a*,*27*,*28*,*29*,*30* sequence. Alphabetizing the letters a–t will produce the standard sequence for *S*. *ochraceum* s. l. (**A**) Basal half of arm (male larva, site 34). (**B**) Terminal half of arm (female larva, site 4). (**C**) Total arm (male larva, site 26, Galapagos Islands). C = centromere.

All populations of cytoform G from the Galapagos Islands, mainland Ecuador, and Puerto Rico were chromosomally cohesive. They differed from the standard banding sequence by 12 fixed inversions, the greatest number for any studied member of the *S*. *ochraceum* complex; all but 2 inversions (*IIIS-1* and *IIIL-14a*) were unique (S1 Table). IIL and IIIL were complexly rearranged (Figs 14 and 15); our hypotheses for deriving them from standard depict 5 fixed inversions each.

We present one of several possible hypotheses for the inversion steps that produced the IIL sequence of cytoform G. The order of the 9 fragments is represented by the letters ‘a’ through ‘p,’ which correspond with the lettering in Fig 14C and 14D. The inversions are assigned the numbers *IIL-14*, *15*, *16*, *17*, and *18*, but the numbers are not indicated below because multiple inversion pathways are possible. The hypothetical inversion breakpoints are indicated below by brackets.

a | kj | de | bc | on **[**gf | lm**]** hi | p = *S*. *ochraceum* cytoform G

a | kj | de | bc **[**onml | fghi**]** p

a **[**kj | de | bc | ihgf**]** lmnop

a **[**fghi | cb**]** ed | jklmnop

abc **[**ihgfed**]** jklmnop

abcdefghijklmnop = *S*. *ochraceum* standard

The following interpretation represents our hypothesis for the 5 inversion steps that produced the IIIL sequence of cytoform G. The scrambled arm, relative to the standard, has 11 fragments. The order of the fragments is represented by the letters ‘a’ through ‘t,’ corresponding with the lettering in Fig 15A and 15B; brackets indicate the inversion that is numbered above each sequence. The order of inversions was constrained by *IIIL-14a*, which is also present in other cytoforms, and, therefore, was considered the first inversion to have occurred in IIIL during the evolutionary derivation of cytoform G from the hypothetical ancestor represented by the standard sequence.

IIIL-27

a **[**hi | on | de | rs | lm | cb**]** gf | qp | jk | t

IIIL-29

abc | ml **[**sr | ed | no | ihgf | qp | jk**]** t

IIIL-30

abc | mlkj **[**pq | fghi**]** on | de | rst

IIIL-28

abc | mlkjihgf **[**qpon | de**]** rst

IIIL-14a

abc **[**milkjihgfed**]** nopqrst

abcdefghijklmnopqrst = *S*. *ochraceum* standard

**Fig 16:**
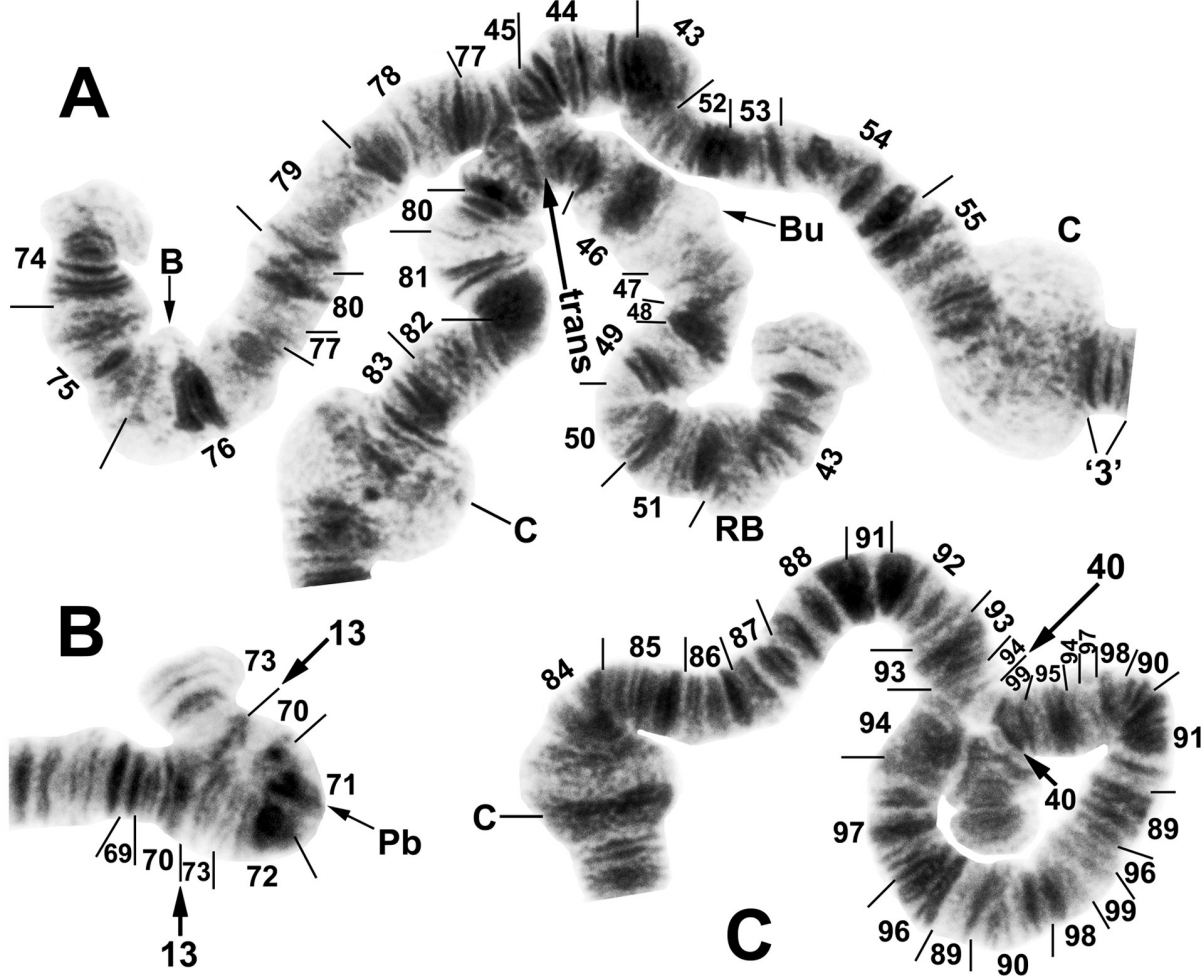
Polymorphisms in *Simulium ochraceum* cytoform G from the Galapagos Islands (female larvae). (**A**) IIS–IIIS translocation heterozygote (site 32). B = blister, Bu = bulge, C = centromere, RB = ring of Balbiani, trans = translocation breakpoint, ‘3’ = 3 sharp. (**B**) IIL end showing IIL-13 heterozygote (site 29). Pb = parabalbiani. (**C**) IIIL arm showing IIIL-40 heterozygote (site 31); arrows indicate breakpoints on the inverted homologue. Section numbering corresponds to the IIIL sequence of cytoform G in Fig 15.

We interpreted *IIS-18* (Fig 12A) as a mimic inversion of *IIS-7*, differing by a few fine bands and perhaps the edge of the ring of Balbiani. In this scenario, *IIS-18* apparently cleaved off the edge of the ring of Balbiani at the 51/43 junction and placed it at the 52/43 junction (Fig 12A). The centromere bands were diffuse (Figs 12A and 15). Only three different autosomal polymorphisms (as heterozygotes) were encountered (Fig 16), all in the Galapagos Islands. IIL-13 occurred in 1 female and 1 male larva from site 29, and IIIL-40 occurred in 1 female larva from site 31 out of 169 analyzed larvae from the Galapagos. A IIS–IIIS heterozygous translocation in all polytene nuclei of 1 female was found at site 32, representing the only known example of an autosomal translocation polymorphism in the Simuliidae.

**Fig 17:**
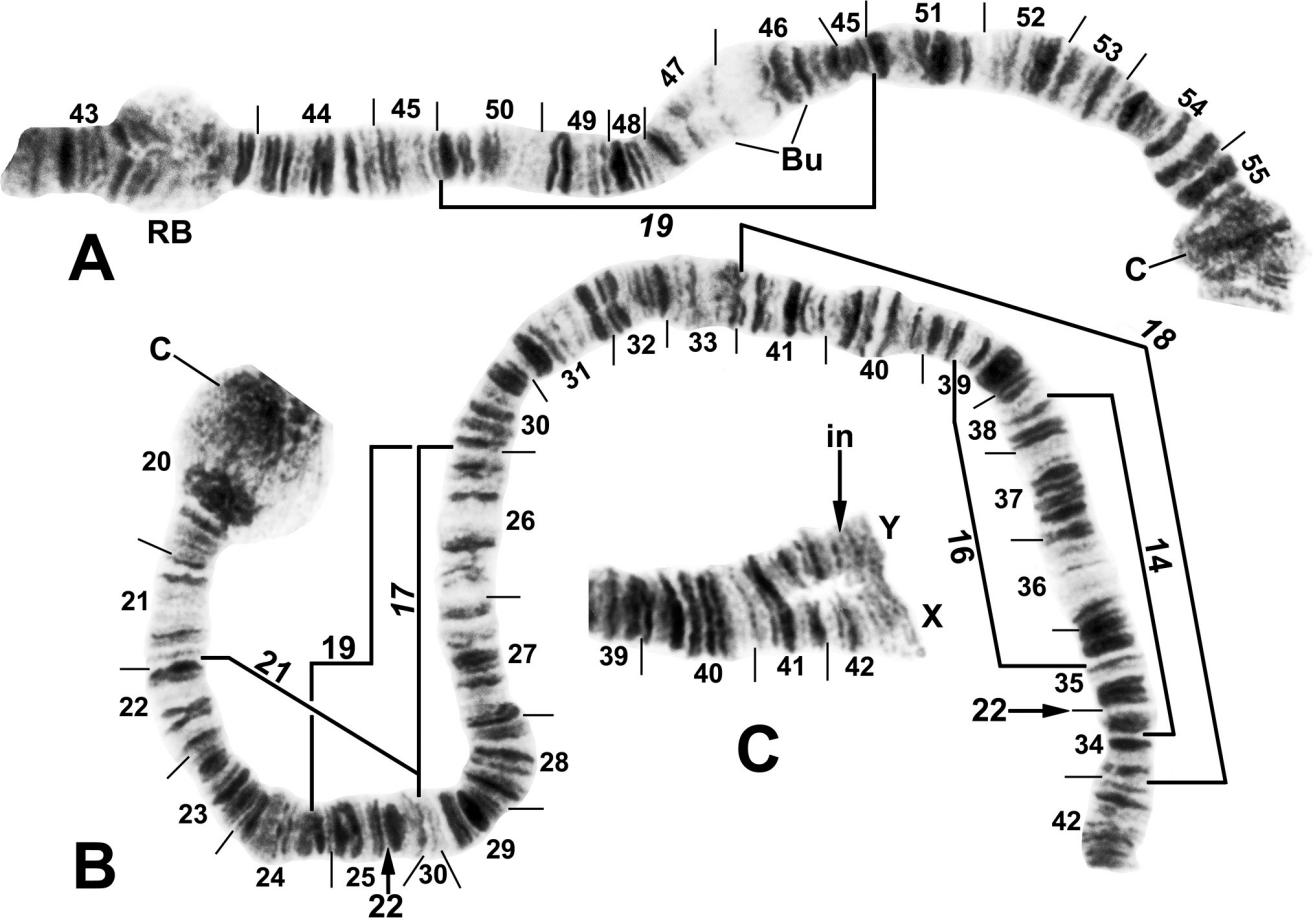
IL and IIS chromosome arms of *Simulium ochraceum* s. l. (**A** and **B**) Cytoform K (female larva, site 34). (**A**) IIS arm showing *IIS-19* sequence. Bu = bulge, C = centromere, RB = ring of Balbiani. (**B**) IL arm showing the *IL-17*,*18* sequence. Other autosomal inversions of *S*. *ochraceum* s. l. are indicated by brackets or arrows. (**C**) Cytoform G male (site 4), showing the IL end with asynaptic terminus and the fine, nearly imperceptible Y-linked supernumerary band insertion (in) indicated by arrow; the supernumerary band is absent in the X homologue.

Cytoform G was one of several cytoforms with differentiated sex chromosomes (Table 2). A nearly imperceptible sex-linked rearrangement was expressed near the end of IL (Fig 17C). In all populations, a slight but consistent asynaptic tendency occurred in the telomeric region of all males, coupled with a subterminal, fine supernumerary band insertion, difficult to discern in suboptimal preparations. The fine supernumerary band and repulsion in the telomeric region were absent in females. The consistency of this difference between males and females suggests that the sex locus for cytoform G is located subterminally in IL.

**Fig 18:**
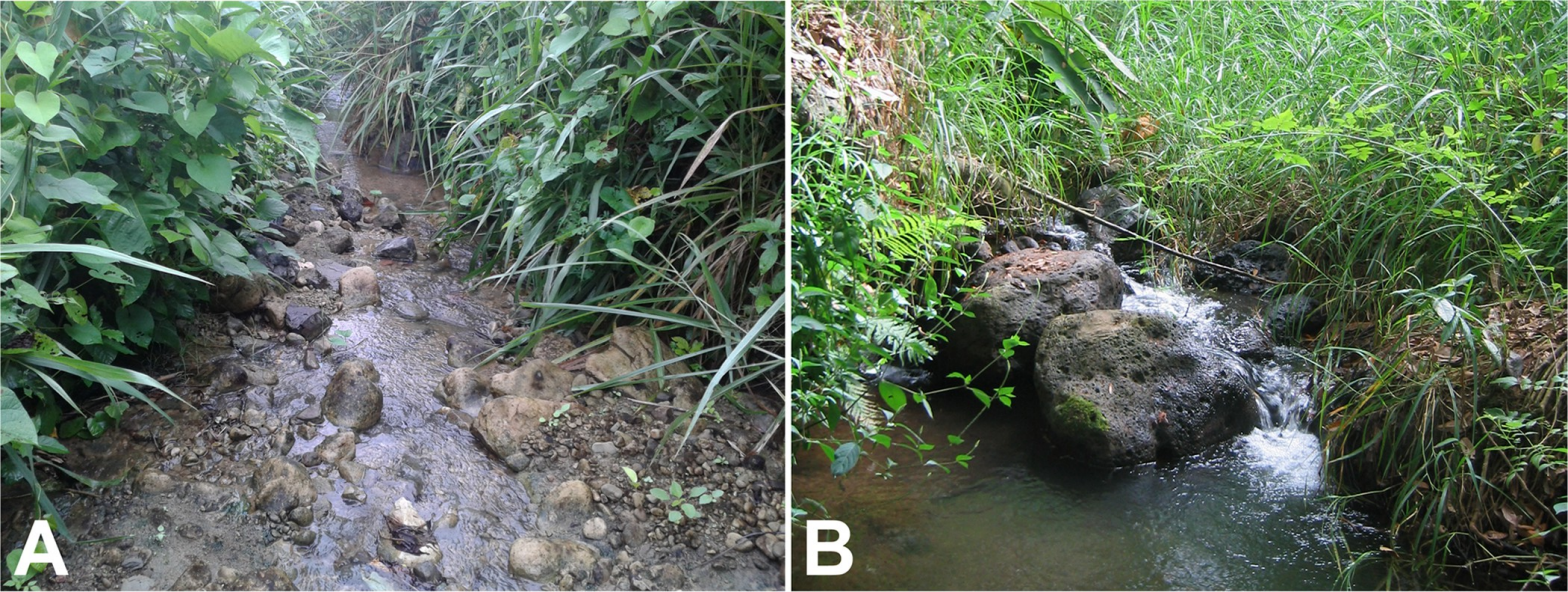
Habitats of larvae and pupae of cytoform G in Ecuador. (**A**) Galapagos, San Cristobal Island (site 31), 22 May 2014. (**B**) Esmeraldas Province (site 8), 20 May 2014. Photos by the authors.

The unique chromosomal banding pattern and sex chromosomes, lowland habitats (43–530 m asl, Fig 18), and morphology, particularly the strong negative head-spot pattern, indicate reproductive isolation of cytoform G from other segregates in our study and the consistency of populations in mainland Ecuador, the Galapagos Islands, and Puerto Rico. None of the 622 larvae (including 169 analyzed chromosomally) collected from the Galapagos Islands were patently infected with mermithid nematodes or other macroscopic parasites and pathogens. Nor were larvae of G from the Ecuadorian mainland or Puerto Rico visibly infected.

### Cytoform H

The second most frequently collected taxon on mainland Ecuador was cytoform H, common among upland streams 567 to 1164 m asl. It was the only cytoform other than E3 that was parasitized with mermithid nematodes (S1 Table). Cytoform H had two unique fixed inversions (S1 Table): *IL-20*, overlain on the *IL-17*,*18* sequence (Fig 3A) of cytoform K, and *IIS-9* (Fig 8A and 8B). It shared *IS-22* (Fig 3B) with cytoform I and *IIL-7* and *IIIL-14a* with 2 and 5 other cytoforms, respectively (plus both with cytoform C [25]). Of the two autosomal polymorphisms, IIS-10 (Fig 8A) was infrequent (0.09) and IIIS-1 (Fig 7A), which was either entirely standard or entirely fixed in all other cytoforms, was in high frequency (0.83). The centromere bands were diffuse (Figs 8A and 13B).

Cytoform H was one of two taxa with B chromosomes. Two larvae (1 female and 1 male; site 21) had a single B chromosome each (Fig 8D). Its heterochromatinized nature suggested that the B chromosome of H was unique vis à vis the B chromosome of cytoform D.

Cytoform H carried a unique set of sex-linked inversions in IIIL on top of fixed inversion *IIIL-14a* (Fig 13B). We defined the sex chromosomes of H relative to the standard sequence for *S*. *ochraceum*. Of the 3 X chromosomes, X_3_ (IIIL-31,32,33) predominated (94.1%). The basic Y sequence in all males was standard for IIIL-32 but combined with other sequences to produce 4 different Y chromosomes (Table 3).

**Table 3:** Sex chromosomes of *Simulium ochraceum* cytoform H in Esmeraldas Province, Ecuador.

Sex-chromosome classes^a^					
X_1_X_3_	X_3_X_3_	X_2_Y_3_	X_3_Y_1_	X_3_Y_2_	X_3_Y_4_
2	16	1	8	5	1

^a^ X_1_ = IIIL-31; X_2_ = IIIL-31,32; X_3_ = IIIL-31,32,33; Y_1_ = IIIL-31,33; Y_2_ = IIIL-31,33,35; Y_3_ = IIIL-31,33,34,35; Y_4_ = IIIL-33,35.

Of 7 streams where H was found, 6 also harbored cytoform E3. No hybrids were found, indicating reproductive isolation of E3 and H.

### Cytoform I

Consisting of only 1 male larva from Ecuador’s Esmeraldas Province at 1156 m asl (site 15), cytoform I had three unique fixed inversions—*IIS-11* (Fig 4A), *IIIL-36*, and *IIIL-37* (Fig 10B)—four shared fixed inversions (*IS-22*, *IL-17*, *IL-18*, and *IIIL-14a*), and unique polymorphisms IIS-12 (Fig 4A) and CI_d_, which we tentatively consider autosomal, although larger sample sizes would allow a test for sex-linkage (S1 Table). CI was heterozygous for a well-defined band and a diffuse band (CI_d_) within a flocculent region associated with unpairing on either side. CII and CIII were well defined. Cytoform I shared IL-17 and IL-18 with cytoforms H and K. Cytoform I was found in the same stream with cytoform E3, from which it differed by 10 fixed inversions. Although represented by a single larva, its unique chromosomal characteristics suggest that it is reproductively isolated, not only from E3, but also from all other studied cytoforms in Ecuador.

### Cytoform J

Represented by 1 male and 1 female larva (without associated pupae or adults) at an elevation of 1341 m in Ecuador’s Esmeraldas Province (site 17), cytoform J was chromosomally unique. Of its 8 fixed inversions, 5 were cytoform specific (S1 Table): *IS-21* (Fig 3C), *IIIL-38*, *IIIL-39* (Fig 6C and 6D), *IIIS-5*, and *IIIS-6* (Fig 11A). *IL-17* and *IIL-7* were shared with 3 other cytoforms, and *IIIS-1* (Fig 7) was shared with 5 others. CI was diffuse (Fig 3C), whereas CII and CIII were well defined (Fig 11A). No polymorphisms or sex-chromosome differentiation were discovered in the small sample. The 2 larvae were found in the same stream with cytoform E3, from which they differed by 10 fixed inversions, indicating reproductive isolation.

Cytoform K (*Simulium dinellii* (Joan))

Our Ecuadorean samples of this cytoform differed from the standard chromosomal sequence by four fixed inversions (S1 Table): *IL-17*, *IL-18*, *IIS-19* (Fig 17A and 17B), and *IIIL-14a*. Sex chromosomes were cytologically undifferentiated, centromere bands typically were diffuse (Fig 17B), and polymorphisms (IL-19) were rare (< 0.01). After establishing K as a distinct cytoform, we assigned the existing name *S*. *dinellii* by comparing reared males and females from sites 6 and 10 with published descriptions and illustrations [45].

### Morphological correlates

Although larval color and head-spot pattern are frequently related to larval sex [46], we found no sexual dimorphism in our material. Larvae of cytoform D were weakly banded, and the head spots varied from slightly positive to slightly negative or imperceptible. Larvae of the E group, especially E3, were strongly banded, the gray bands contrasting with unpigmented (whitish) areas; the uninterrupted terminal band covering abdominal segments V–VIII was prominent. The head-spot pattern of the E group was typically indiscernible. Reared adults from sites that supported larvae exclusively or predominantly of E3 had silver vittae on the orange scutum. Reared adults were not available for E1 or E2. Larvae of F were not distinguishable from those of E2 at the same site. Other life stages of F were not available for study. Mature larvae of G were the largest larvae in our study and the only larvae with uniformly distributed pigment on the body and a well-defined negative head-spot pattern, which was consistent in populations from mainland Ecuador, the Galapagos Islands, and Puerto Rico. Larvae of H in acetic ethanol could be sorted consistently from E3 by their faint bluish, rather than gray, pigment distributed more uniformly over the body and a faint negative head-spot pattern. Reared males, possibly of this cytoform, had the silver scutal vittae characteristic of the *S*. *ochraceum* complex. Insufficient material of I and J was available to characterize the larvae, although they were initially sorted morphologically as members of the E group. Reared adults from sites pure for cytoform K allowed us to link the chromosomal characterization with the name *S*. *dinellii*. These adults were the only ones in our material that did not have silver vitae on the orange scutum.

## Discussion

### Taxonomic status of cytoforms

Taxonomy matters. The case of *S*. *ochraceum* s. l. offers a clear example of the first principle of successful pest management and conservation biology: accurate identification of the target species [5,47]. The high frequency of cryptic species among most groups of organisms [48,49] provides the lesson that a fine level of taxonomy with integrated methodological approaches [50] is essential if we are to make appropriate decisions when considering population suppression or eradication of a species, particularly in insular systems.

Coupled with the three cytoforms recognized in earlier studies [24,25], *S*. *ochraceum* s. l. now comprises 13 morphologically similar cytoforms. Each cytoform is defined by a unique chromosomal profile, and about half of the cytoforms have uniquely differentiated sex chromosomes. Ecologically, the cytoforms can be divided into a lowland (< 500 m asl) group (E1, E2, F, and G) and a highland (> 500 m asl) group (A, B, C, D, E3, H, I, and J), with cytoform K appearing in both groups. We suggest that at least the following nine cytoforms are full species, inferred from an absence of hybrids in sympatry or quasisympatry, ecological characteristics (e.g., lowland vs. upland), geographic distributions, and unique structural (primarily larval) features: A, C, E3, F, G, H, I, J, and K. The greatest aerial distance between any two Ecuadorian cytoforms in our study is 135 km (between F and I).

The species status of *S*. *dinellii*, originally based on morphological criteria and, until 1985, considered conspecific with *S*. *bipunctatum* [51], is chromosomally confirmed. It is anthropophilic, biting the arms and face [45], although we encountered no biting or nuisance swarming in Esmeraldas Province. We suspect that *S*. *dinellii* is a species complex, given its broad geographic range—coast to coast and from Venezuela south to Argentina [15]. We, therefore, retain our *a priori* cytoform designation, ‘K’, for *S*. *dinellii*, anticipating eventual discovery of additional cytoforms of this nominal taxon.

Among nominal taxa closely related to *S*. *ochraceum*, we expected that *S*. *shewellianum* Coscarón might be represented in our material, having been described originally from Ecuador, and characterized as a small-stream inhabitant at about 400 m asl [45]. However, none of our pupae or adults match the original description. The published coordinates for the type locality—Rio Palenque (0°35’S 70°2W [*sic*]) [45]—are corrupted, placing the type locality on the eastern fringe of Colombia, whereas Rio Palenque (Los Rios Province) in Ecuador is a few hundred kilometers south of Esmeraldas Province. Among other possible nominal species in our material, *S*. *nuneztovari* Ramírez-Pérez, Rassi & Ramírez was not represented among cytoforms for which we had link-reared adults (E3, G, H, and K), given that its adults lack the silver scutal vittae [52]. The location of the type locality of *S*. *nuneztovari* in Cacurí, Venezuela, about 1500 km to the east of Esmeraldas Province and on the opposite side of the Andes, also reduces the probability that it might be represented among our cytoforms.

Cytoforms A and B differ from one another in their sex chromosomes and autosomal polymorphism profiles, and C differs from A and B by five fixed inversions, unique sex chromosomes, and cytoform-specific autosomal polymorphisms [25]. A and C are known from Mexico and Guatemala, and cytoform B only from Mexico. The evidence at hand indicates that *S*. *ochraceum* sensu stricto (= cytoform A) does not reach South America. Its confirmed southern limit is Guatemala [25], although we would expect it in similar habitats at least somewhat southward. Although A and C are reproductively isolated [25], the specific status of B, vis à vis A, cannot be judged, given the absence of fixed-inversion differences in allopatry (ca. 600 km apart). B might simply be a chromosomal variant (cytotype) of A.

Cytoform D is most closely related to C, but given their allopatric nature, about 770 aerial km apart, their taxonomic status relative to one another cannot be resolved. The probable location of the sex locus on different chromosomes (IIL in D vs IIIL in C) provides support for separate species. Nonhomologous sex chromosomes typically reflect different species [53]. Regardless, at least one of the two cytoforms is a distinct, but formally unnamed, species.

The E group provides an example of the challenge of assessing taxonomic status of cytoforms across geographic gaps, in this case gaps of 45–118 km. The specific status of the three segregates is unknown, whether a single polymorphic species, multiple species, or species *in statu nascendi*. Each cytoform represents an elevational set of populations: E2 at 56–150 m asl, E1 at 169–331 m asl, and E3 at 567–1341 m asl. Elevation is a defining attribute among cytoforms of the *S*. *ochraceum* complex in Central America [25] and is a probable isolating mechanism during speciation in other simuliids [54]. Sampling geographically intermediate sites is needed to resolve the status of E1, E2, and E3.

Cytoform G (from the Galapagos Islands, mainland Ecuador, and Puerto Rico) is chromosomally the most remote of the 13 segregates in our study—12 to 19 fixed inversions removed from all others. Its relationships within the *S*. *ochraceum* complex, however, are obscure. Its nearly monomorphic chromosomes, coupled with the same sex-chromosome rearrangement across all screened populations, suggest a single widespread species.

Accepting the available evidence that G is a single widespread species, what is its formal identity? The nomenclatural history of the Galapagos black flies provides a window into the uncertainty of a formal identity. Upon its discovery, the Galapagos population was called *S*. *bipunctatum* [12]. It was later referred to, depending on the authority, as *S*. *ochraceum* [17] or *S*. *antillarum* [45], with *S*. *ochraceum* being the more widely used name [22]. It clearly, however, is not *S*. *ochraceum* sensu stricto, which doubtfully even reaches South America. We are, thus, presented with three reasonable possibilities: *S*. *antillarum*, *S*. *bipunctatum*, and *S*. *wolcotti*. Having twice sampled all flowing water on St. Croix (Virgin Islands), the type locality of *S*. *antillarum*, we infer that no black flies currently exist on the island. However, given the proximity (ca. 100 km) of the type locality of *S*. *antillarum* to that of *S*. *wolcotti* (Puerto Rico), we agree with the original morphology-based proposal [55] that *wolcotti* is a junior synonym of *antillarum*. Of particular relevance to the formal identity of G is *S*. *bipunctatum*, whose identity hinges on the holotype from Peru. Charles H. T. Townsend collected numerous insects along the Rio Charape in Jaén Province of northern Peru [56], including the female specimen on 13 November 1911, which would become the holotype of *S*. *bipunctatum*. Although we can reasonably assign the name *antillarum* and its synonym, *wolcotti*, to G via the chromosomal similarity of Puerto Rican and Ecuadorian material, we are not able to assign the name *bipunctatum* to a cytoform because of lack of chromosomal material from the type locality. If the name *biunctatum* applies to G, then by the principle of priority in the International Code of Zoological Nomenclature, it would become the formal name of the Galapagos entity, predating the name *antillarum* by three years. Based on available evidence, we revalidate the name *antillarum* for populations of G in Puerto Rico, mainland Ecuador, and the Galapagos Islands.

In agreement with our chromosomal results, COI-barcoding studies show no divergence between *S*. *ochraceum* s. l. from San Cristobal on the Galapagos Islands and in Esmeraldas Province (Cayapa River) [30,57]. The San Cristobal + Esmeraldas clade is the sister group of material from near the type locality of *S*. *bipunctatum* in Peru, and both of these clades are the sister group of specimens from the type locality (Cayey, Puerto Rico) of *S*. *wolcotti* [30,57]. Whether the entire clade can be viewed as multiple species or as a single species that would take the oldest available name (*S*. *bipunctatum*), with the subclades merely reflecting locational differences, must await analysis of geographically intervening collections. In the COI tree, the entire clade, however, is separate and well removed from a clade containing *S*. *ochraceum* from Mexico (including the type locality) and Costa Rica [30,57].

Of the remaining four cytoforms, F is a lowland taxon (95 m asl) ecologically distinct from H, I, and J, which are upland taxa (565–1335 m asl). Cytoform H is on strongest footing for species status, given its larger sample size (33 larvae) across multiple streams (7), in addition to its unique chromosomal attributes. Despite the limited samples (one or two) of cytoforms I and J, we consider each a distinct species reproductively isolated at least from cytoforms E3, H, and one another, based on minimal intervening geographic distances (< 4 km). The possibility that one or more of these four cytoforms is simply a variant of another cytoform in our study compounds improbabilities that they each would be homozygous for multiple unique inversions.

The presence of nine members of the *S*. *ochraceum* complex in a small, geographically circumscribed area (ca. 6,000 km^2^) of mainland Ecuador (Esmeraldas Province) suggests that additional diversity exists in the complex in other geographic areas, particularly east of the Andean cordillera. In Brazil, for instance, *S*. *ochraceum* s. l. has been recorded from streams less than 1 m wide to 20 m wide [22], hinting at the possibility of multiple species [58], probably including taxa not yet discovered among the cytoforms in our study.

### The Galapagos black fly—native or adventive?

The Neotropical mainland holds a large pool of simuliid species—nearly 400 nominal species [15]. Yet the only simuliid ever recorded from the Galapagos Islands is cytoform G, to which we have formally assigned the name *S*. *antillarum*. The distance of nearly 1000 km between the mainland and the islands presents a formidable barrier to arrival of simuliid propagules [59]. The barrier is so great for insects that natural colonization of the archipelago from the mainland is estimated to be about one insect species every 2000 years [60]. On the other hand, the colonization rate for introduced insects on the Galapagos was estimated in 2006 to be about one species per year since the first recorded visit by humans in 1535 [61]; the rate is now higher [9].

As further testament to open ocean as a barrier for simuliids, no unequivocally documented case is known of a black fly deliberately or unintentionally introduced to an island, with one possible exception. *Simulium aureohirtum* Brunetti, the only non-native species in Oceania, was first recorded on Guam in 2009 and is chromosomally and structurally identical to mainland populations, suggesting a recent arrival, although via an unknown mode [62]. The case for recent arrival is further supported by the absence of *S*. *aureohirtum* in previous surveys of black flies on the island [63]. Nearly all simuliids on remote oceanic islands—those 500 km or more from a source population—have diverged markedly in structure and typically represent precinctive species and higher taxa including, *inter alia*, the genus *Crozetia* in the Crozet Islands and the subgenera *S*. (*Hebridosimulium*) in Vanuatu and *S*. (*Inseliellum*) in French Polynesia [64–66].

Successful colonization of the Galapagos Islands by black flies would depend not only on surviving the long-distance dispersal, but also on discovery by the founding female(s) of suitable habitat for the immature stages. Females subsequently would typically need an acceptable blood source to mature their eggs. No species of black fly is known to feed on blood hosts other than birds and mammals; the giant tortoises, iguanas, and other herpetofauna, therefore, would be irrelevant to blood-feeding black flies. The hosts of *S*. *ochraceum* s. l. and related species on the mainland are primarily mammals but are not well known beyond humans and farm animals (e.g., cattle, horses, and chickens). Cattle, donkeys, goats, horses, and pigs were introduced to San Cristobal about 1847, cats perhaps in the early settlement period, dogs in the mid-1800s, black rats (*Rattus rattus* L.) in the mid- to late 1800s, Norway rats (*R*. *norvegicus* Berkenhout) in the 1980s, and domesticated birds such as chickens in the mid-1900s into the 2000s [67]. We question whether the majority of available hosts (primarily birds) before the 1840s would have been adequate for a species presumed to be primarily mammalophilic. The few originally available, potential mammalian hosts might have included the Galapagos fur seal (*Arctocephalus galapagoensis* Heller) and Galapagos sea lion (*Zalophus wollebaeki* Sivertsen).

The presence of *S*. *antillarum* on the Galapagos Islands, in addition to the Caribbean islands more than 3000 km from the Galapagos—much of this distance over open water—suggests that the species is an adept colonizer with a tendency for females to disperse well beyond their natal streams. Further evidence of its dispersal abilities was documented after the rainfall of the 1997–1998 El Niño enabled the species to occupy temporary flows on the Galapagos Islands of Floreana, Isabela, and Santiago [10].

The precise source of the Galapagos population is unknown. The nearly monomorphic chromosomes of *S*. *antillarum* minimize the use of polymorphic rearrangements to pinpoint the source area. With more sampling, however, the rare Galapagos IIL-13 and IIIL-40 polymorphisms and perhaps the translocation polymorphism (if capable of persisting) might be found on the mainland, assuming that they are mainland-derived and not *de novo* archipelago rearrangements.

Given 8 to 16 generations per year for *S*. *antillarum* on San Cristobal, which is probable for tropical simuliids including *S*. *ochraceum* s. l. [68], we estimate that about 200 to 400 generations were completed from the time black flies were first discovered on the island in 1989 to when our collections were made in 2014. The estimated number of island generations is probably conservative, given that colonization probably occurred sometime before black flies reached population levels on the Galapagos that would have been noticed. Some degree of genetic change and adaptation to the island habitat since colonization might reasonably be expected. Macrogenomic (cytogenetic) changes have been documented in one of North America’s premier simuliid colonizers, *Simulium vittatum* Zetterstedt. After 18 years (ca. 140 generations) of laboratory colonization, a statistically significant change was found in the degree of sex linkage of inversions and in the frequency of the most common autosomal polymorphisms [69]. Thus, the rare polymorphisms in the Galapagos population could have been derived on the islands in the past several decades. Alternatively, they might have originated via one or more colonizations of the Galapagos Islands from one or more polymorphic populations on the mainland. The polymorphic molecular loci reported in the Galapagos population [14] could similarly be argued.

Independent evidence for the source of black flies on the Galapagos Islands might be sought in their DNA or associated symbiotic community. Molecular genetic markers have helped define source areas for invasions of agricultural and public health pests [70–72]. The existence of a large, diverse community of bacteria in larval and adult black flies [73] suggests the possibility of a “symbiote fingerprint”. The only recorded symbiote in Galapagos black flies, however, is an unidentified species of the trichomycete fungal genus *Smittium*, which inhabits the larval hind guts [14]. Some *Smittium* species, however, tend to be less family-specific than other trichomycete fungi [74], and thus might have arrived on the Galapagos Islands in another host, such as a chironomid midge or mosquito. Without a more specific identification, the utility of this trichomycete as an indicator of a source population is limited. Although larvae of mainland cytoforms E3 and H had mermithid nematodes, those of *S*. *antillarum* from all examined populations were free of patent infections with pathogens or parasites.

We are in no better position than were previous workers to speculate on the mode of dispersal to the islands, whether via airplane, in a shipment of bananas, by wind-powered flight, or otherwise [12]. Regardless of whether the dispersal was natural or via human agency, our survey of Esmeraldas Province indicates that a source population is opportunely located near the Pacific coast of South America in the general area with the shortest distance (ca. 1000 km) to the Galapagos Islands. Travel and commerce are routinely conducted from Guayaquil, which lies about 275 km south of our Esmeraldas collection sites for *S*. *antillarum*. Historically, particularly during the World War II occupation of Baltra (ca. 90 km northwest of San Cristobal) by the US military, air traffic was extraordinarily high from mainland stations in Ecuador and Central America [75].

On balance, all evidence indicates that the black flies in the Galapagos Islands are a single species—*Simulium antillarum*—conspecific with mainland populations and widespread in the Neotropical Region. The unique chromosomal banding pattern, novel sex chromosomes, lowland habitats of the immature stages, and characteristic larval morphology demonstrate the consistency of studied populations in mainland Ecuador, the Galapagos Islands, and Puerto Rico. Other than polymorphic molecular loci [14] and perhaps the rare chromosomal polymorphisms, little evidence supports anything other than a recent arrival, whether through human agency or natural dispersal. Despite the presence of humans, including a steady flow of naturalists, collectors, and writers on San Cristobal since Darwin disembarked there in 1835 [76], we are not aware of any reference to a black fly or even to a biting insect that could reasonably be conceived as a black fly until 1989 [12]. An alternative interpretation holds that agricultural development and construction of a reservoir, with the potential to increase larval abundance, could have unmasked a much older presence of *S*. *antillarum* on San Cristobal [14].

Any management considerations for *S*. *antillarum* in the Galapagos, including the possibility of eradication from the islands, should consider its current ecological role in the aquatic and terrestrial environment [77]. *Simulium antillarum* might, for instance, have assumed a functional role in the streams of San Cristobal, either by occupying an available niche or by displacing or otherwise affecting native lotic organisms. In the terrestrial landscape of the Galapagos Islands, the females are major biting pests of humans and domesticated animals [10,11], but any tendency to feed on the native animals or transmit agents of disease is unknown. Despite their pestiferous nature, black flies are not readily detected when blood-feeding on wildlife [77]. At the very least, biologists should be aware of the potential for *S*. *antillarum* to feed on the native birds and mammals, particularly the larger mammals, such as Galapagos fur seal and Galapagos sea lion.

### Biting and disease risks

Despite its broad geographic distribution, *S*. *ochraceum* s. l. is highly anthropophilic only in restricted areas of its total range, *viz*., Brazil (Upper Amazon), Galapagos Islands, Guadeloupe, Guatemala, and Mexico [10,16,22,78]. Biting by *S*. *ochraceum* s. l. has not been recorded for Puerto Rico, nor did we experience anthropophily in mainland Ecuador. Where it is anthropophilic, its vectorial role is restricted to former onchocerciasis foci in Guatemala and Mexico [20] inhabited by cytoforms A (= true *S*. *ochraceum*), B, and C [27]. Its non-vector status in other New World onchocerciasis foci [20] might reflect its zoophilic nature, an important factor influencing the extent of the disease burden associated with the *S*. *damnosum* complex in African onchocerciasis areas [49]. *Simulium ochraceum* s. l. was not implicated as a vector in the Santiago onchocerciasis focus of Esmeraldas Province where it is primarily zoophilic [21]. Morphological evidence indicates that cytoform G (i.e., *S*. *antillarum*) was present in the Santiago onchocerciasis focus. The larva illustrated from the Santiago focus [21] corresponds to cytoform G, the only cytoform in our study with a well-defined negative head-spot pattern (*cf*. figure 125 of [21]). Other than its association with human onchocerciasis, *S*. *ochraceum* s. l. has not been implicated in any vector-borne disease of vertebrates, although screening has been minimal.

The discrepancy between intense anthropophily of *S*. *antillarum* in the Galapagos Islands and probable zoophily of the same species in Esmeraldas Province is not presently reconcilable. Varying degrees of anthropophily across areas is known in other chromosomally cohesive black flies, such as *S*. *venustum* Say in North America [46]. We suggest, however, that for *S*. *antillarum* the phenomenon relates, at least in part, to population size. Streams in the Galapagos Islands produce large populations of flies [13]. In contradistinction, our entire collection effort in mainland Ecuador—two or three collectors sampling each of 46 sites for 30–45 minutes over 9 days—recovered only 79 larvae of *S*. *antillarum*. Pest status of simuliids depends, in large part, on the ability to reach substantial population levels [1,79]. Our experience with *S*. *ochraceum* s. l. indicates that it typically occurs in small populations. Certainly, that is the case throughout Esmeraldas Province. The small size of mainland streams, typically less than 2 m wide, in which the immature stages develop, limits fly abundance, even though *S*. *ochraceum* s. l. occupies most small, shaded streams. Where these streams are common, such as on the Pacific slopes of the Sierra Madre in Guatemala, *S*. *ochraceum* s. l. is said to “abound” [16]. On San Cristobal Island, with a human population of about 7,200 people [80], the streams have experienced changes in water flow and have increased in productivity as a result of human settlement and agricultural runoff. These conditions promote increased fly populations, abetted by the depauperate fauna of the Galapagos streams, which present few predation risks and little competition from other macroinvertebrates and none from other simuliid species.

## Conclusions

Of the 13 cytoforms now known in the *S*. *ochraceum* complex, only cytoform G is found on the Galapagos Islands. Cytoform G, to which the name *S*. *antillarum* is logically applied, is also found in mainland Ecuador and the Caribbean. It is, therefore, not precinctive to the Galapagos. Whether it is native (predating human contact with the Galapagos) or adventive (arriving more recently) cannot be determined based on available evidence.

## Supporting information

S1 Table Frequency of rearrangements in chromosomal homologues of the *Simulium ochraceum* complex. (DOCX)
