# Supplementary material for: Black flies (Diptera: Simuliidae) in the Galapagos Islands: Native or adventive?
Source: PLoS One. 2024 Oct 24;19(10):e0311808. doi: 10.1371/journal.pone.0311808 (PMC11501040; doi:10.1371/journal.pone.0311808)
Supplement: S1 Table — (DOCX) [file pone.0311808.s001.docx]

**S1 Table. Frequency of rearrangements in chromosomal homologues of the *Simulium ochraceum* complex.**

| Cytoform | D | D | E1 | E2 | E3 | F |
| --- | --- | --- | --- | --- | --- | --- |
| Provenance (Site No.) | Costa Rica (1) | Panama (33) | Ecuador (7, 25) | Ecuador (4, 5, 8, 9) | Ecuador (10–19, 21, 23)^a^ | Ecuador (5) |
| F:M^c^ | 7:5 | 4:1 | 3:4 | 11:10 | 48:39 | 3:8 |
| IS-16 | 0.04 |  |  |  |  |  |
| IS-17 | 0.08 | 0.60 |  |  |  |  |
| IS-18 |  |  |  |  | <0.01 |  |
| IS-19 | 0.96 | 1.00 |  |  |  |  |
| IS-20 |  |  |  |  | 0.02 |  |
| *IS-21* |  |  |  |  |  |  |
| *IS-22* |  |  |  |  |  |  |
| IS-23 |  |  | 0.07 |  | <0.01 |  |
| IS N.O.^d^ |  |  |  |  | <0.01 |  |
| IL-14 | 0.04 |  |  |  |  |  |
| IL-15 |  |  |  |  |  |  |
| IL-16 |  | 0.10 |  |  |  |  |
| *IL-17* |  |  |  |  |  |  |
| *IL-18* |  |  |  |  |  |  |
| IL-19 |  |  |  |  |  |  |
| *IL-20* |  |  |  |  |  |  |
| IL-21 |  |  |  |  | <0.01 |  |
| IL-22 |  |  |  |  | 0.01 |  |
| IL hc36^e^ |  |  |  |  | 0.01 |  |
| IL 2°N.O.^f^ |  |  | 0.07 |  |  |  |
| IL telomere^g^ |  |  |  |  |  |  |
| IL in42^h^ |  |  |  |  |  |  |
| CI_d_^j^ |  |  |  |  |  |  |
| *IIS-7* | 1.00 | 1.00 |  |  |  |  |
| *IIS-8* | 1.00 | 1.00 |  |  |  |  |
| *IIS-9* |  |  |  |  |  |  |
| IIS-10 |  |  |  |  |  |  |
| *IIS-11* |  |  |  |  |  |  |
| IIS-12 |  |  |  |  |  |  |
| IIS-13 | 0.79 | 0.50 |  |  |  |  |
| IIS-14 | 0.58 |  |  |  |  |  |
| *IIS-15* |  |  | 1.00 | 1.00 | 1.00 |  |
| *IIS-16* |  |  |  |  |  | 1.00 |
| *IIS-17* |  |  |  |  |  | 1.00 |
| *IIS-18* |  |  |  |  |  |  |
| *IIS-19* |  |  |  |  |  |  |
| IIS–IIIS trans^k^ |  |  |  |  |  |  |
| *IIL-7* | 1.00 | 1.00 |  |  |  |  |
| IIL-8 |  |  |  |  |  |  |
| IIL-9 | *^i^ | * |  |  |  |  |
| IIL-10 | * | * |  |  |  |  |
| IIL-11 | * |  |  |  |  |  |
| IIL-12 | * |  |  |  |  |  |
| IIL-13 |  |  |  |  |  |  |
| *IIL-14* |  |  |  |  |  |  |
| *IIL-15* |  |  |  |  |  |  |
| *IIL-16* |  |  |  |  |  |  |
| *IIL-17* |  |  |  |  |  |  |
| *IIL-18* |  |  |  |  |  |  |
| IIL hyb56^l^ | * | 1.00^m^ |  |  |  |  |
| IIIS-1 |  |  | 1.00 | 1.00 | 1.00 |  |
| IIIS-2 | 0.96 | 1.00 |  |  |  |  |
| IIIS-3 |  |  |  |  | <0.01 |  |
| IIIS-4 |  |  |  |  | 0.01 |  |
| *IIIS-5* |  |  |  |  |  |  |
| *IIIS-6* |  |  |  |  |  |  |
| IIIS in83^n^ | 0.04 |  |  |  |  |  |
| *IIIL-12a* | 1.00 | 1.00 |  |  |  |  |
| *IIIL-13a* | 1.00 | 1.00 |  |  |  |  |
| *IIIL-14a* | 1.00 | 1.00 |  |  |  | 1.00 |
| *IIIL-15a* | 1.00 | 1.00 |  |  |  |  |
| IIIL-18 | 0.33 |  |  |  |  |  |
| IIIL-19 |  | 0.20 |  |  |  |  |
| *IIIL-20* |  |  | 1.00 | 1.00 | 1.00 |  |
| IIIL-21 |  |  | 1.00 | 1.00 | 0.93 |  |
| IIIL-22 |  |  |  |  | 0.54 |  |
| IIIL-23 |  |  | 0.93 | 1.00 | 0.53 |  |
| IIIL-24 |  |  |  | 0.93 |  |  |
| IIIL-25 |  |  |  |  | 0.11 |  |
| IIIL-26 |  |  |  | 0.02 |  |  |
| *IIIL-27* |  |  |  |  |  |  |
| *IIIL-28* |  |  |  |  |  |  |
| *IIIL-29* |  |  |  |  |  |  |
| *IIIL-30* |  |  |  |  |  |  |
| IIIL-31 |  |  |  |  |  |  |
| IIIL-32 |  |  |  |  |  |  |
| IIIL-33 |  |  |  |  |  |  |
| IIIL-34 |  |  |  |  |  |  |
| IIIL-35 |  |  |  |  |  |  |
| *IIIL-36* |  |  |  |  |  |  |
| *IIIL-37* |  |  |  |  |  |  |
| *IIIL-38* |  |  |  |  |  |  |
| *IIIL-39* |  |  |  |  |  |  |
| IIIL-40 |  |  |  |  |  |  |
| IIIL-cmpx^o^ | 0.04 |  |  |  |  |  |
| CIII_e_^p^ |  |  | 0.07 |  |  |  |
| Bs^q^ | 0.08 |  |  |  |  |  |
| Mean no. of heterozygous autosomal inversions/larva | 2.00 | 1.80 | 0.14 | 0.10 | 1.36 | 0.00 |

**S1 Table, continued. Cytoforms G through K.**

| Cytoform | G | G | G | H | I | J | K |
| --- | --- | --- | --- | --- | --- | --- | --- |
| Provenance (Site No.) | Ecuador, Galapagos (26–32) | Ecuador (3, 4, 8) | Puerto Rico (34–37) | Ecuador (10–13, 21^b^, 23, 24) | Ecuador (15) | Ecuador (17) | Ecuador (2, 6, 10, 20, 22, 25) |
| F:M^c^ | 99:70 | 24:15 | 24:21 | 18:15 | 0:1 | 1:1 | 35:33 |
| IS-16 |  |  |  |  |  |  |  |
| IS-17 |  |  |  |  |  |  |  |
| IS-18 |  |  |  |  |  |  |  |
| IS-19 |  |  |  |  |  |  |  |
| IS-20 |  |  |  |  |  |  |  |
| *IS-21* |  |  |  |  |  | 1.00 |  |
| *IS-22* |  |  |  | 1.00 | 1.00 |  |  |
| IS-23 |  |  |  |  |  |  |  |
| IS N.O.^d^ |  |  |  |  |  |  |  |
| IL-14 |  |  |  |  |  |  |  |
| IL-15 |  |  |  | 0.01 |  |  |  |
| IL-16 |  |  |  |  |  |  |  |
| *IL-17* |  |  |  | 1.00 | 1.00 | 1.00 | 1.00 |
| *IL-18* |  |  |  | 1.00 | 1.00 |  | 1.00 |
| IL-19 |  |  |  |  |  |  | < 0.01 |
| *IL-20* |  |  |  | 1.00 |  |  |  |
| IL-21 |  |  |  |  |  |  |  |
| IL-22 |  |  |  |  |  |  |  |
| IL hc36^e^ |  |  |  |  |  |  |  |
| IL 2°N.O.^f^ |  |  |  |  |  |  |  |
| IL telomere^g^ |  | 0.01 |  |  |  |  |  |
| IL in42^h^ | *^i^ | * | * |  |  |  |  |
| CI_d_^j^ |  |  |  |  | 0.50 |  |  |
| *IIS-7* |  |  |  |  |  |  |  |
| *IIS-8* |  |  |  |  |  |  |  |
| *IIS-9* |  |  |  | 1.00 |  |  |  |
| IIS-10 |  |  |  | 0.09 |  |  |  |
| *IIS-11* |  |  |  |  | 1.00 |  |  |
| IIS-12 |  |  |  |  | 0.50 |  |  |
| IIS-13 |  |  |  |  |  |  |  |
| IIS-14 |  |  |  |  |  |  |  |
| *IIS-15* |  |  |  |  |  |  |  |
| *IIS-16* |  |  |  |  |  |  |  |
| *IIS-17* |  |  |  |  |  |  |  |
| *IIS-18* | 1.00 | 1.00 | 1.00 |  |  |  |  |
| *IIS-19* |  |  |  |  |  |  | 1.00 |
| IIS–IIIS trans^k^ | <0.01 |  |  |  |  |  |  |
| *IIL-7* |  |  |  | 1.00 |  | 1.00 |  |
| IIL-8 |  |  |  | 0.01 |  |  |  |
| IIL-9 |  |  |  |  |  |  |  |
| IIL-10 |  |  |  |  |  |  |  |
| IIL-11 |  |  |  |  |  |  |  |
| IIL-12 |  |  |  |  |  |  |  |
| IIL-13 | <0.01 |  |  |  |  |  |  |
| *IIL-14* | 1.00 | 1.00 | 1.00 |  |  |  |  |
| *IIL-15* | 1.00 | 1.00 | 1.00 |  |  |  |  |
| *IIL-16* | 1.00 | 1.00 | 1.00 |  |  |  |  |
| *IIL-17* | 1.00 | 1.00 | 1.00 |  |  |  |  |
| *IIL-18* | 1.00 | 1.00 | 1.00 |  |  |  |  |
| IIL hyb56^l^ |  |  |  |  |  |  |  |
| IIIS-1 | 1.00 | 1.00 | 1.00 | 0.83 |  | 1.00 |  |
| IIIS-2 |  |  |  |  |  |  |  |
| IIIS-3 |  |  |  |  |  |  |  |
| IIIS-4 |  |  |  |  |  |  |  |
| *IIIS-5* |  |  |  |  |  | 1.00 |  |
| *IIIS-6* |  |  |  |  |  | 1.00 |  |
| IIIS in83^n^ |  |  |  |  |  |  |  |
| *IIIL-12a* |  |  |  |  |  |  |  |
| *IIIL-13a* |  |  |  |  |  |  |  |
| *IIIL-14a* | 1.00 | 1.00 | 1.00 | 1.00 | 1.00 |  | 1.00 |
| *IIIL-15a* |  |  |  |  |  |  |  |
| IIIL-18 |  |  |  |  |  |  |  |
| IIIL-19 |  |  |  |  |  |  |  |
| *IIIL-20* |  |  |  |  |  |  |  |
| IIIL-21 |  |  |  |  |  |  |  |
| IIIL-22 |  |  |  |  |  |  |  |
| IIIL-23 |  |  |  |  |  |  |  |
| IIIL-24 |  |  |  |  |  |  |  |
| IIIL-25 |  |  |  |  |  |  |  |
| IIIL-26 |  |  |  |  |  |  |  |
| *IIIL-27* | 1.00 | 1.00 | 1.00 |  |  |  |  |
| *IIIL-28* | 1.00 | 1.00 | 1.00 |  |  |  |  |
| *IIIL-29* | 1.00 | 1.00 | 1.00 |  |  |  |  |
| *IIIL-30* | 1.00 | 1.00 | 1.00 |  |  |  |  |
| IIIL-31 |  |  |  | * |  |  |  |
| IIIL-32 |  |  |  | * |  |  |  |
| IIIL-33 |  |  |  | * |  |  |  |
| IIIL-34 |  |  |  | * |  |  |  |
| IIIL-35 |  |  |  | * |  |  |  |
| *IIIL-36* |  |  |  |  | 1.00 |  |  |
| *IIIL-37* |  |  |  |  | 1.00 |  |  |
| *IIIL-38* |  |  |  |  |  | 1.00 |  |
| *IIIL-39* |  |  |  |  |  | 1.00 |  |
| IIIL-40 | <0.01 |  |  |  |  |  |  |
| IIIL-cmpx^o^ |  |  |  |  |  |  |  |
| CIII_e_^p^ |  |  |  |  |  |  |  |
| Bs^q^ |  |  |  | 0.06 |  |  |  |
| Mean no. of heterozygous autosomal inversions/larva | 0.02 | 0.00 | 0.00 | 0.52 | 2.00 | 0.00 | 0.01 |

^a^ The following larvae of cytoform E3 were parasitized by unidentified mermithid nematodes: 2 females and 1 male (sites 11 and 15); 1 female (sites 12 and 21).

^b^ One female larva of cytoform H was parasitized by an unidentified mermithid nematode.

^c^ Female larvae: male larvae.

^d^ Heterozygous expression of the primary nucleolar organizer (N.O.) in 1 male larva (site 12).

^e^ Heterochromatic insertion IL hc36 occurred on the same homologue with IL-22 in 1 male and 1 female larva (site 14).

^f^ Secondary nucleolar organizer (N.O.) in section 35 of IL in 1 female larva (site 25).

^g^ Telomere heterozygously thickened in 1 male larva (site 3).

^h^ Supernumerary fine band insertion (in) in section 42 of IL in all males.

^i^ * = Sex linked.

^j^ Centromere band heterozygously diffuse and flocculent in 1 male.

^k^ IIS–IIIS translocation (trans) heterozygously expressed in 1 female larva (site 32).

^l^ hyb = heavy band in section 56C2.

^m^ hyb (heavy band) is tentatively considered fixed in the Panama sample, based on its expression in 10 of 10 chromosomal homologues (as opposed to sex-linked in the Costa Rica sample).

^n^ Supernumerary band insertion (in) in section 83 of IIIS in 1 male larva.

^o^ Breakpoints for a complex (cmpx) set of inversions in the middle two-thirds of IIIL of 1 female from Costa Rica were not resolved.

^p^ Centromere band heterozygously enhanced in 1 male larva (site 7).

^q^ Frequency of larvae with B chromosomes.
